# Circular RNA circIKBKB promotes breast cancer bone metastasis through sustaining NF-κB/bone remodeling factors signaling

**DOI:** 10.1186/s12943-021-01394-8

**Published:** 2021-07-29

**Authors:** Yingru Xu, Shuxia Zhang, Xinyi Liao, Man Li, Suwen Chen, Xincheng Li, Xingui Wu, Meisongzhu Yang, Miaoling Tang, Yameng Hu, Ziwen Li, Ruyuan Yu, Mudan Huang, Libing Song, Jun Li

**Affiliations:** 1 Program of Cancer Research, Key Laboratory of Protein Modification and Degradation and Guangzhou Institute of Oncology, Affiliated Guangzhou Women and Children’s Hospital, School of Basic Medical Sciences, Guangzhou Medical University, Guangzhou, 510623 China; 2 Department of Biochemistry, Zhongshan School of Medicine, Sun Yat-Sen University, Guangzhou, 510080 China; 3 State Key Laboratory of Oncology in South China, Collaborative Innovation Center for Cancer Medicine, Sun Yat-Sen University Cancer Center, Guangzhou, 510080 China

**Keywords:** Bone metastasis, Breast cancer, circIKBKB, Bone remodeling factor, NF-κB

## Abstract

**Background:**

Breast cancer (BC) has a marked tendency to spread to the bone, resulting in significant skeletal complications and mortality. Recently, circular RNAs (circRNAs) have been reported to contribute to cancer initiation and progression. However, the function and mechanism of circRNAs in BC bone metastasis (BC-BM) remain largely unknown.

**Methods:**

Bone-metastatic circRNAs were screened using circRNAs deep sequencing and validated using in situ hybridization in BC tissues with or without bone metastasis. The role of circIKBKB in inducing bone pre-metastatic niche formation and bone metastasis was determined using osteoclastogenesis, immunofluorescence and bone resorption pit assays. The mechanism underlying circIKBKB-mediated activation of NF-κB/bone remodeling factors signaling and EIF4A3-induced circIKBKB were investigated using RNA pull-down, luciferase reporter, chromatin isolation by RNA purification and enzyme-linked immunosorbent assays.

**Results:**

We identified that a novel circRNA, circIKBKB, was upregulated significantly in bone-metastatic BC tissues. Overexpressing circIKBKB enhanced the capability of BC cells to induce formation of bone pre-metastatic niche dramatically by promoting osteoclastogenesis in vivo and in vitro. Mechanically, circIKBKB activated NF-κB pathway via promoting IKKβ-mediated IκBα phosphorylation, inhibiting IκBα feedback loop and facilitating NF-κB to the promoters of multiple bone remodeling factors. Moreover, EIF4A3, acted acting as a pre-mRNA splicing factor, promoted cyclization of circIKBKB by directly binding to the circIKBKB flanking region. Importantly, treatment with inhibitor eIF4A3-IN-2 reduced circIKBKB expression and inhibited breast cancer bone metastasis effectively.

**Conclusion:**

We revealed a plausible mechanism for circIKBKB-mediated NF-κB hyperactivation in bone-metastatic BC, which might represent a potential strategy to treat breast cancer bone metastasis.

**Supplementary Information:**

The online version contains supplementary material available at 10.1186/s12943-021-01394-8.

## Background

Female breast cancer (BC) has become the leading cause of global cancer incidence worldwide [1]. The majority of BC fatalities are due to complications from recurrent or tumor metastases in distant organs [2, 3]. Thereinto, the most common site of BC metastasis is the bone, occurring in 70% of patients with metastatic BC [4, 5]. Currently, the clinical interventions for patients with BC-bone metastasis (BC-BM) are bisphosphonates (BPs) or denosumab (an anti-receptor activator of nuclear factor kappa B ligand (RANKL) monoclonal antibody), which disrupt the vicious cycle by targeting osteoclasts [6]. However, these drugs have no effect on the patient survival rate and also cause severe side effects, even leading to BC metastasis to visceral organs [7–9]. Therefore, understanding the mechanisms of BC-BM is essential to develop innovative therapeutic strategies and improve of patient outcomes.

BC-BMs mostly are osteolytic metastases that are characterized by aberrant bone destruction caused by increased osteoclasts-mediated bone resorption in the tumor-bone interface [10]. Accumulating evidence has proven that cancer-secreted factors play direct or indirect roles in the activation of osteoclasts, i.e., to absorb the bone matrix in bone compartments, which establishes a “bone pre-metastatic niche” to support cancer bone metastasis [5, 9, 11–13]. In turn, the degraded bone matrix released various bone matrix-bound growth factors that benefit the seeding and expansion of metastatic tumor cells in bone, which forming a “vicious cycle” [9].

Hyperactivation of nuclear factor kappa B (NF-κB) signaling has been reported to be involved in cancer bone-metastasis via upregulation of multiple bone-remodeling factors, such as RANKL, parathyroid hormone-related protein preproprotein (PTHrP), macrophage colony stimulating factor (M-CSF) and granulocyte–macrophage colony stimulating factor (GM-CSF) [14–18]. Systemically blocking NF-κB activity via IκB kinase (IKK) inhibitors or nondegradable NF-κB inhibitor alpha (IκBα) dramatically inhibited osteolytic bone metastasis of BC in vivo [18]. However, systemic inhibition of NF-κB activity might also promote tissue injury via inducing cell apoptosis and could even promote hepatocarcinogenesis by inducing reactive oxygen species (ROS) accumulation and liver damage [19, 20]. Therefore, investigating the mechanism of NF-κB hyperactivation in BC would provide effective target for BC-BM treatment.

Circular RNAs (circRNAs) are a class of widespread circular RNAs that are covalently closed single stranded circular transcripts formed by precursor mRNA back-splicing or skipping events, which have no 5′ caps or 3′ poly(A) tail [21]. Clinical evidence and animal studies have demonstrated that aberrant expression of circRNAs contribute to carcinogenesis and development of BC [22–24]. In the present study, we identified a novel circRNA, circIKBKB (hsa_circ_0084100), derived from the IKBKB gene (encoding inhibitor of NF-κB kinase subunit beta), which plays an important role in promotion of osteoclastogenesis and BC-BM through inducing bone pre-metastatic niche formation via specific upregulation of multiple bone remodeling factors. Importantly, treatment with an inhibitor of eukaryotic translation initiation factor 4A3 (EIF4A3), the circIKBKB cyclization factor, reduced circIKBKB expression and effectively inhibited BC-BM. Taken together, our results uncover a plausible mechanism underlying the intrinsic metastatic property of BC to bone and might represent an attractive therapeutic target in the treatment of BC-BM.

## Methods

### Patient information

This study, which complied with all relevant ethical regulations for work with human participants, was conducted on a total of 20 tumor-adjacent normal breast tissues and 331 paraffin-embedded BC samples, including 295 primary BC tissues (237 non-BM/BC and 58 BM/BC) and 36 bone-metastatic BC tissues (at bone), that were histopathologically and clinically diagnosed at the Third Affiliated Hospital and Sun Yat-sen University Cancer Center from 2005 to 2018. The study protocols were approved by the Institutional Research Ethics Committee of Sun Yat-sen University for the use of these clinical materials for research purposes. All Patients’ samples were obtained according to the Declaration of Helsinki and each patient signed a written informed consent for all the procedures.

### RNA sequencing of circRNA extracted from human BC tissues

The total RNA was extracted from six BC (without BM) and six BC (with BM) tissues using TRIzol reagent (Takara, Dalian, China) and further purified by rRNA depletion, followed by cDNA synthesis and RNA amplification, according to the manufacturer’s instructions. The RNA-seq libraries were constructed and sequenced utilizing the Illumina HiSeq2500 platform (Illumina, San Diego, USA).

### ASO-mediated knockdown and ASO in vivo treatment

The antisense oligonucleotides (ASOs) targeting circIKBKB were obtained from RiboBio (Guangzhou, China). Transfections with ASOs (50 nM) were performed with Lipofectamine RNAiMAX according to the manufacturer’s instructions. RNA and protein were harvested for analysis 72 h after transfection. For in vivo treatment experiment, 10 nmol ASOs were delivered into mouse through tail vein injection twice a week.

### Xenografted tumor models

All of the animal procedures were approved by the Sun Yat-sen University Animal Care Committee. Intracardiac injections of luciferase-expressing BC cells (1 × 10^5^) were conducted in nu/nu nude mice (5 weeks old) for bone metastasis assays. IKK-I VI (1 mg/kg, iv) or eIF4A3-IN-2 (1 mg/kg, po) were injected twice per day. The formation of bone metastases was observed and assessed weekly in mice injected with D-luciferin (75 mg/kg) by bioluminescence imaging using the IVIS Spectrum In Vivo Imager. At week 5, the osteolytic lesions in mice were observed using SIEMENS micro-CT system (Inveon). The bone of mice was harvested and prepared for further analyses.

### Statistical analysis

Statistical analysis was performed using the Student’s two-tailed t-test and One-way analysis of variance (ANOVA). Bivariate correlations between study variables were calculated by Spearman’s rank correlation coefficients. Survival curves were plotted by the Kaplan–Meier method and compared by the log-rank test. The significance of various variables for survival was analyzed by univariate and multivariate Cox regression analyses. *P*-values of 0.05 or less were considered statistically significant. All data were presented as the mean ± standard deviation (SD). Statistical analysis was performed using the GraphPad Prism 7 and SPSS 19.0 statistical software. Representation of the *P*-values was *P < 0.05, **P < 0.01, ***P < 0.001, and *ns*: not significant (*P* > 0.05).

More method details (see Additional file 4).

## Results

### Overexpression of circIKBKB induces osteolytic bone metastasis of breast cancer

**Fig. 1:**
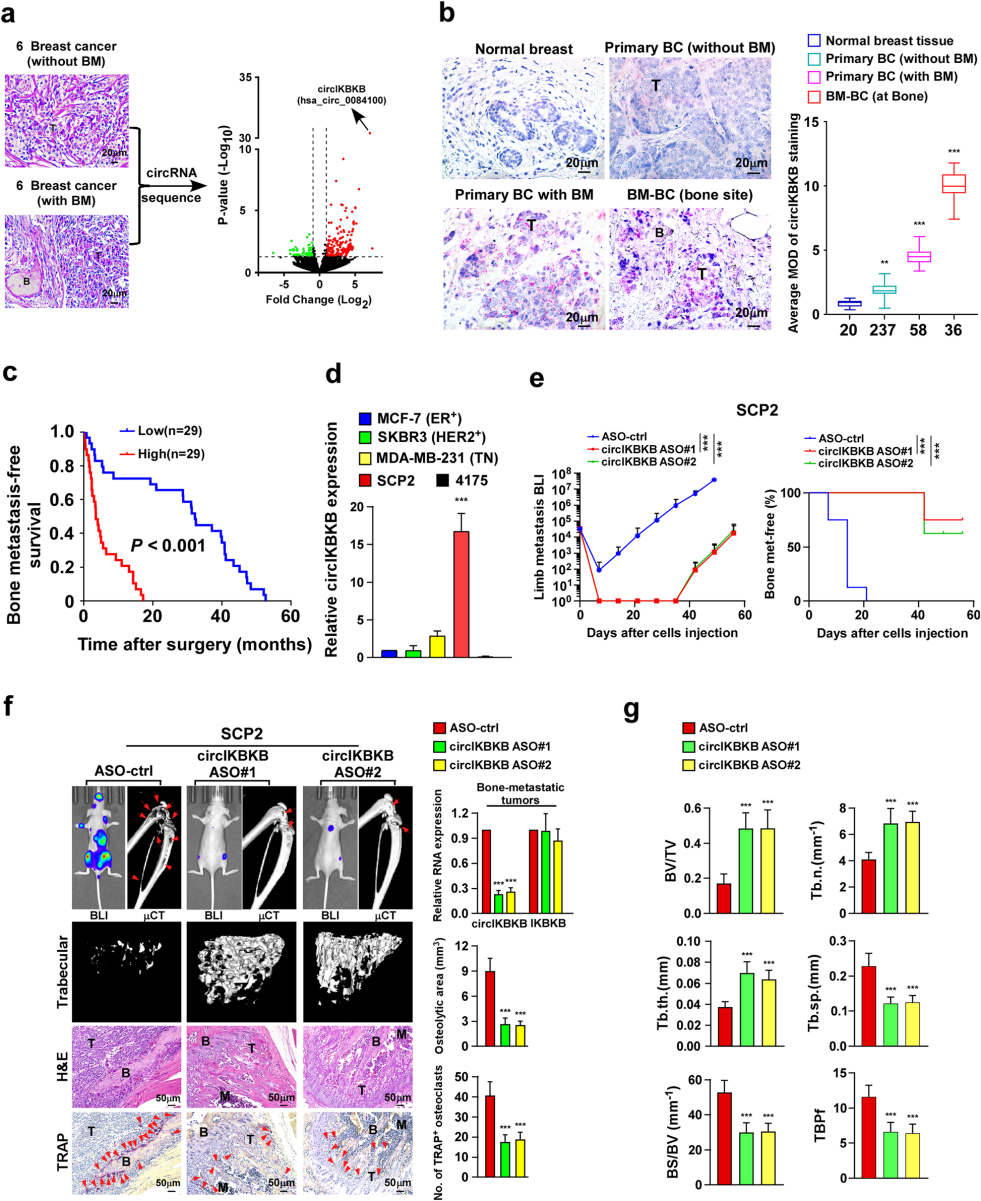
Silencing circIKBKB inhibits osteolytic bone metastasis of breast cancer in vivo. **a** H&E analysis (left) and Volcano plot analysis (right) of dysregulated circRNAs from circRNAs deep sequencing comparing BC tissues with or without bone metastasis (*n* = 6). **b** ISH analysis (left) and quantification (right) of circIKBKB expression in 20 normal breast tissues and 331 clinical BC tissues, including 295 primary BC tissues (237 non-BM/BC and 58 BM/BC) and 36 bone-metastatic BC tissues (at bone). **c** Kaplan–Meier analysis of bone metastasis-free survival curves in patients with BM/BC with low vs high expression of circIKBKB (*n* = 58; *P* < 0.001, log-rank test). **d** Real-time PCR analysis of circIKBKB expression in the indicated cells. GAPDH served as a loading control. **e** Normalized BLI signals of bone metastases and Kaplan–Meier bone metastasis-free survival curve of mice from the indicated experimental groups (*n* = 8/group). **f** Left: BLI, μCT (longitudinal and trabecular section) and histological (H&E and TRAP staining) images of bone lesions from representative mice. Scale bar, 50 μm. Right: Quantification of circIKBKB and IKBKB expression and μCT osteolytic lesion area and TRAP^+^ osteoclasts along the bone-tumor interface of metastases from experiment in the left panel. **g** Quantification of bone parameters from representative mice in (**g**). BV/TV, bone/tissue volume ratio; BS/TV, bone surface/ tissue volume ratio; Tb. n, trabecular number; Tb. sp., trabecular separation; Tb. th., trabecular thickness; TBPf, trabecular bone pattern factor. Each error bar represents the mean ± SD of three independent experiments. * *P* < 0.05, ** *P* < 0.01, *** *P* < 0.001

To identify the critical circRNAs that contribute to BC-BM, circRNA deep-sequencing was performed in six primary BC tissues without BM (non-BM/BC) and six bone-metastatic BC tissues (BM/BC) (Fig. 1a). Comparative analysis revealed that a total of 214 circRNAs were dysregulated, including 163 significantly upregulated and 51 downregulated circRNAs, in BM/BC tissues compared with non-BM/BC tissues (Fig. 1a and Table S1). According to circBase (http://www.circbase.org/), we found that the most significantly upregulated circRNA, circRNA hsa_circ_0084100, was derived from exon 3-4-5 of the IKBKB transcript, named as circIKBKB hereafter (Fig. S1a).

We then further examined the circular characteristics of circIKBKB. As shown in Fig. S1a-e, sanger sequencing indicated that the back-splice junction of circIKBKB was from exon 5 to exon 3 of IKBKB, reverse transcription PCR (RT-PCR) analyses using convergent and divergent primers showed that circIKBKB was only amplified by divergent primers from cDNA but not from gDNA. Using random or oligo DT primers indicated circIKBKB had no poly-A tail. In addition, circIKBKB was more stable than linear IKBKB mRNA upon RNase-R/actinomycin D treatment.

The expression of circIKBKB was then examined using in situ hybridization (ISH), with a specific probe targeting the junction sequence of circIKBKB, in 20 normal breast tissues and 331 clinical BC tissues, including 295 primary BC tissues (237 non-BM/BC and 58 BM/BC) and 36 bone-metastatic BC tissues (at bone). As shown in Fig. 1b, circIKBKB signals were undetectable in normal breast tissues, and only marginally detectable in primary non-BM/BC tissues but increased in primary BM/BC tissues (with BM) and were elevated markedly in bone-metastatic BC tissues (at bone). Importantly, *ISH* statistical analysis revealed that patients with high circIKBKB-expressing BC had significantly shorter bone-metastasis-free survival than those with low circIKBKB-expressing BC (*P* < 0.001; Fig. 1c). Taken together, these results suggest that circIKBKB overexpression correlates with BC-BM.

To determine the role of circIKBKB in BC-BM, we investigated the effect of silencing circIKBKB by using antisense oligonucleotide (ASO) targeting the circIKBKB junction sequence, on bone-metastasis of SCP2 cells, which is a bone-tropic breast cancer cell line with high circIKBKB expression compared to low bone-metastatic breast cancer cell lines, including MCF7, SKBR3, MDA-MB-231 and 4175 (Fig. 1d). The quantitative real-time PCR (qRT-PCR) showed that circIKBKB-ASO treatment significantly reduced expression of circIKBKB, but not IKBKB mRNA, in bone-metastatic tumors (Fig. 1f). Prominently, compared to ASO-control treated mice, circIKBKB-ASO treatment resulted in less and delayed incidence of bone metastases and significant reduction of metastasis burden in SCP2 cell-injected mice (Fig. 1e, f). Statistical analysis of micro-computed tomography (μCT) revealed that circIKBKB-ASO-treated mice displayed significantly decreased BM lesions/osteolytic areas, accompanied with relatively increased the volume/number/thickness of trabecular and reduced trabecular separation and bone pattern factor (Fig. 1g). Importantly, we found that circIKBKB-ASO treatment significantly reduced the tartrate-resistant acid phosphatase (TRAP)^+^-osteoclasts along the bone-tumor interface compared to those in control mice (Fig. 1f). Taken together, these results indicate that silencing circIKBKB inhibits BC-BM.

### CircIKBKB overexpression promotes osteolytic bone metastasis of breast cancer in vivo

**Fig. 2:**
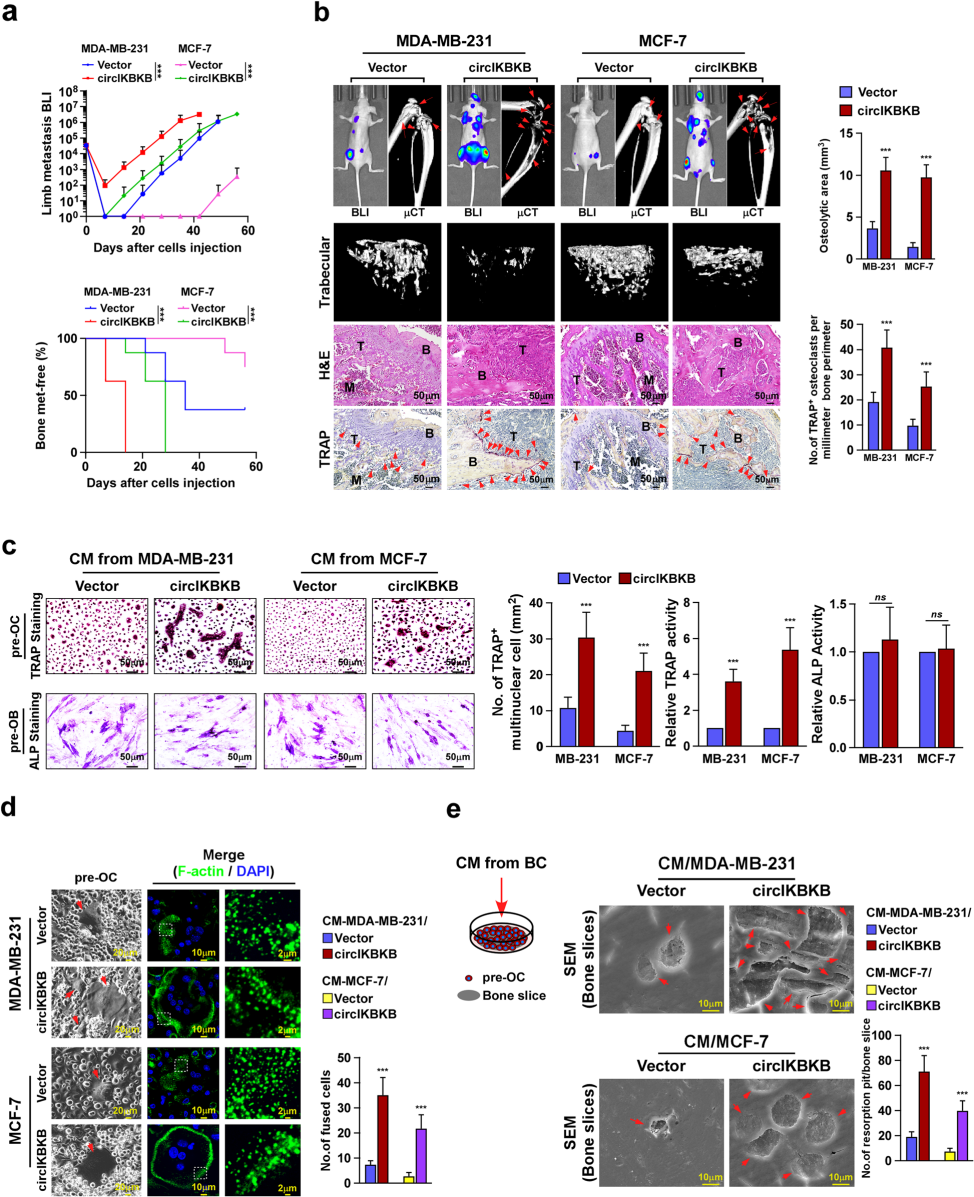
Overexpression of circIKBKB promotes osteolytic bone metastasis of breast cancer in vivo. **a** Normalized BLI signals of bone metastases and Kaplan–Meier bone metastasis-free survival curve of mice from the indicated experimental groups (*n* = 8/group). **b** Left: BLI, μCT (longitudinal and trabecular section) and histological (H&E and TRAP staining) images of bone lesions from representative mice. Scale bar, 50 μm. Right: Quantification of μCT osteolytic lesion area and TRAP^+^ osteoclasts along the bone-tumor interface of metastases from experiment in the left panel. **c** Osteoclast differentiation assay by TRAP staining (upper) or osteoblast differentiation assay by ALP staining (lower) in the presence of CM from indicated cells. Right: Quantification of number of TRAP^+^-multinuclear osteoclasts, TRAP activity and ALP activity from experiment in left panel. **d** Left: Phase contrast micrograph of pre-osteoclasts treated with CM from indicated cells (left) and IF staining images of phalloidin (F-actin) (middle and right). Scale Bar, 20 µm (left), 10 µm (middle) and 2 µm (right). Right: Quantification of the number of fused multinuclear cells from experiment in the left panel. **e** Bone resorption assay analysis of pre-osteoclasts cultured onto the bone slices treated with CM from indicated cells (left), then bone slice was fixed for scanning electron microscopy (SEM) (middle) and quantification of the number of resorption pits per bone slice (right). Each error bar represents the mean ± SD of three independent experiments. * *P* < 0.05, ** *P* < 0.01, *** *P* < 0.001

To further investigate the effect of circIKBKB overexpression on BC-BM, BC cell line MCF-7, with low circIKBKB expression, and MDA-MB-231, with moderate circIKBKB expression, were established to stably express circIKBKB and firefly luciferase reporter (Fig. 1d). Ectopically expressing circIKBKB did not alter the level of IKBKB mRNA in these BC cell lines (Fig. S2a). In vivo bone metastasis model monitored by bioluminescence imaging (BLI) showed that the mice intracardially injected with circIKBKB-overexpressing BC cells exhibited earlier bone metastatic onsets and larger bone metastatic tumor-burden (Fig. 2a, b). μCT analysis indicated that, compared to the vector-control mice, circIKBKB/mice displayed larger osteolytic bone lesions together with significant modulation of bone parameters, such as decreased trabecular volume/number/thickness and increased trabecular separation/bone pattern factor (Fig. 2b and Fig. S2b). Meanwhile, we observed dramatically increased numbers of TRAP^+^-osteoclasts represented along the bone-tumor interface in circIKBKB/mice (Fig. 2b), suggesting that circIKBKB-overexpressing BC cells might possess a strong capability to induce the formation of bone pre-metastatic niche.

### Overexpression of circIKBKB in BC cells induces osteoclastogenesis

Consistent with the abovementioned hypothesis, we observed that the numbers of TRAP^+^-multinucleated mature osteoclasts and TRAP enzymatic activity were drastically increased in pre-osteoclasts (pre-OC) treated with conditioned media (CM) from BC/circIKBKB cells (CM-BC/circIKBKB) (Fig. 2c). However, stimulated with CM-BC/circIKBKB had no effect on the differentiation of pre-osteoblasts (pre-OB), as indicated by no obvious change in the number of ALP^+^-osteoblasts and RANKL/OPG ratio (Fig. 2c and Fig. S2c). These results indicate that overexpressing circIKBKB in BC cells induces osteoclastogenesis. Indeed, we found that CM-BC/circIKBKB treatment induced the expression of multiple osteoclastogenesis-related markers, including FBJ osteosarcoma oncogene (*C-fos*), acid phosphatase 5, tartrate resistant (*Acp5*), cathepsin K (*Ctsk*), nuclear factor of activated T cells 1 (*Nfat-c1*) and dendrocyte expressed seven transmembrane protein (*Dc-stamp*), and facilitated the fusion of pre-osteoclasts, accompanying with increased podosome (actin ring) formation (Fig. S2d and Fig. 2d). Importantly, bone resorption assay showed that CM-BC/circIKBKB-treated osteoclasts possessed higher bone-resorbing activity (Fig. 2e), resulting in an elevated the level of bone matrix-released transforming growth factor beta (TGF-β) and consequently promoted BC cells proliferation (Fig. S2e). Taken together, these results indicate that upregulation of circIKBKB in BC cells induces osteoclastogenesis.

### Silencing circIKBKB in BC cells reduces osteoclastogenesis

Consistent with in vivo results that circIKBKB-ASO treatment decreased the number of TRAP^+^-osteoclasts along the bone-tumor interface in SCP2/mice (Fig. 1g), silencing circIKBKB significantly reduced the inductive effect of CM/SCP2 cells on osteoclastogenesis, as indicated by reduced TRAP^+^-multinucleated mature osteoclasts and TRAP enzymatic activity, as well as decreased the expression of osteoclastogenesis-related markers (Fig. S3a-c). Correspondingly, the stimulatory effects of CM/SCP2 on the fusion events and bone-resorbing activity of osteoclasts were abolished by circIKBKB downregulation (Fig. S3d, e). These results provide further evidence that circIKBKB plays a crucial role in induction of osteoclastogenesis.

### Overexpression of circIKBKB activates NF-κB signaling pathway in BC cells

**Fig. 3:**
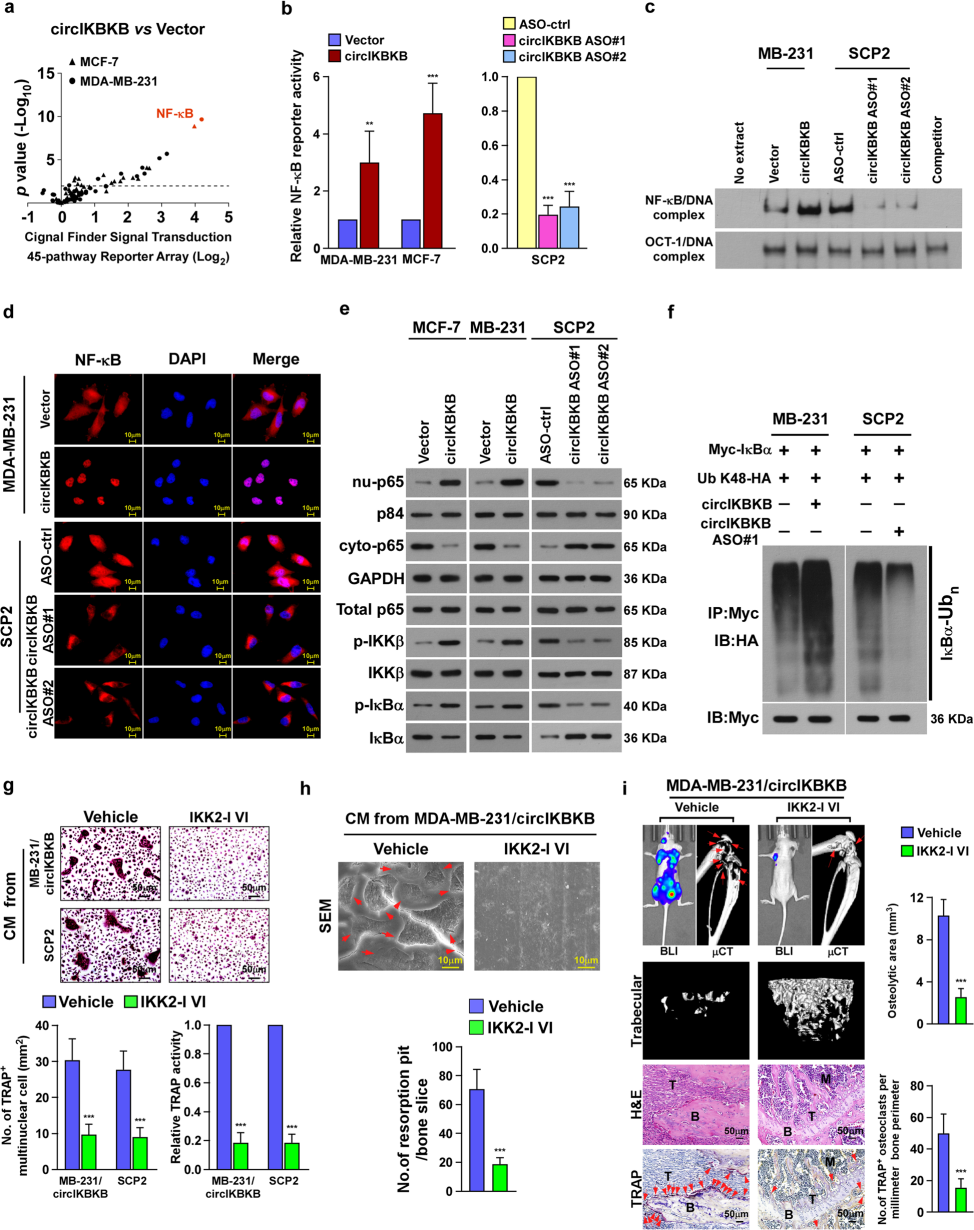
Activation of NF-κB is essential for circIKBKB-induced BC bone-metastasis. **a** Cignal finder signal transduction 45-pathway reporter array showing that circIKBKB overexpression significantly activated NF-κB signaling in BC cells. **b** Relative NF-κB-driven luciferase activity was analyzed in the indicated cells treated with TNF-α (2 ng/ml). **c** EMSA of the endogenous NF-κB activity in the indicated cells. Oct-1/DNA-binding complex was used as a control. **d** Subcellular localization of NF-κB p65 in the indicated cells as analyzed by an immunofluorescence staining assay. **e** Western blotting analysis of level of cytoplasmic-p65, nuclear-p65, total p65, p-IKK-β, total IKK-β, p-IκB-α, and total IκB-α in the indicated cells treated with TNF-α (2 ng/ml). GAPDH served as a cytoplasmic control and p84 served as a nuclear control. **f** Western blotting analysis of the K48-linked polyubiquitin levels of IκB-α in the indicated cells. **g** Upper: Osteoclast differentiation assay by TRAP staining in the presence of CM from vehicle and IKK2-I VI-treated cells. Lower: Quantification of the number of TRAP^+^-multinuclear osteoclasts and TRAP activity from experiment in the upper panel. **h** SEM images (upper) and quantification of the number of resorption pits (lower) of bone slice resorbed by pre-osteoclasts in the presence of CM from vehicle and IKK2-I VI-treated cells. **i** BLI, μCT (longitudinal and trabecular section) and histological (H&E and TRAP staining) images of bone lesions from vehicle- and IKK2-I VI-treated mice. Scale bar, 50 μm. Right: Quantification of μCT osteolytic lesion area and TRAP^+^ osteoclasts along the bone-tumor interface of metastases from experiment in the left panel. Each error bar represents the mean ± SD of three independent experiments. * *P* < 0.05, ** *P* < 0.01, *** *P* < 0.001

To further clarify the mechanism underlying circIKBKB-mediated osteoclastogenesis, cignal finder signal transduction 45-pathway reporter array was performed in vector- and circIKBKB-transduced BC cells. Strikingly, the NF-κB transcriptional activity was most significantly induced in both circIKBKB-overexpressing cells compared to control cells (Fig. 3a), suggesting that circIKBKB might be involved in modulation of NF-κB pathway. In line with this hypothesis, overexpressing circIKBKB also significantly increased, but silencing circIKBKB decreased, the NF-κB-driven luciferase activity, the NF-κB/DNA binding activity, the NF-κB nuclear level and the level of K48-linked polyubiqutinated IκBα (Fig. 3a-f). However, the promotive effect of circIKBKB on NF-κB pathway was drastically abrogated by ectopically expressing a specific NF-κB inhibitor (IκBα-mu) (Fig. S4a). These results suggest that circIKBKB overexpression activates NF-κB pathway in BC cells.

### Blocking NF-κB inhibits circIKBKB-induced osteoclastogenesis and bone metastasis

Next, we examined the contribution of NF-κB signaling to circIKBKB-induced osteoclastogenesis and bone metastasis. We first tested the effect of IKK-β inhibitor VI (IKK2-I VI), which dramatically inhibited the NF-κB pathway in SCP2 and MDA-MB-231/circIKBKB cells (Fig. S4b). In vitro experiments showed that compared to vehicle treatment, IKK2-I VI treatment considerably abolished the inductive effect of CM from SCP2 and MDA-MB-231/circIKBKB cells on osteoclastogenesis and bone-resorbing activity (Fig. 3g-h). Furthermore, we found that IKK2-I VI treatment significantly decreased the incidence of bone metastasis, reduced the size and number of osteolytic bone lesions and lessened the number of TRAP^+^-osteoclasts along the bone-tumor interface (Fig. 3i and Fig. S4c, d). Similar to effect of IKK2-I VI, blocking NF-κB via overexpression of IκBα-mu also showed inhibitory effects on circIKBKB-induced osteoclastogenesis and bone metastasis (Fig. S4e-h). Therefore, these data demonstrate that NF-κB signaling activation is essential for circIKBKB-induced osteoclastogenesis and bone metastasis.

### CircIKBKB activates NF-κB pathway both in cytoplasma and nuclear

**Fig. 4:**
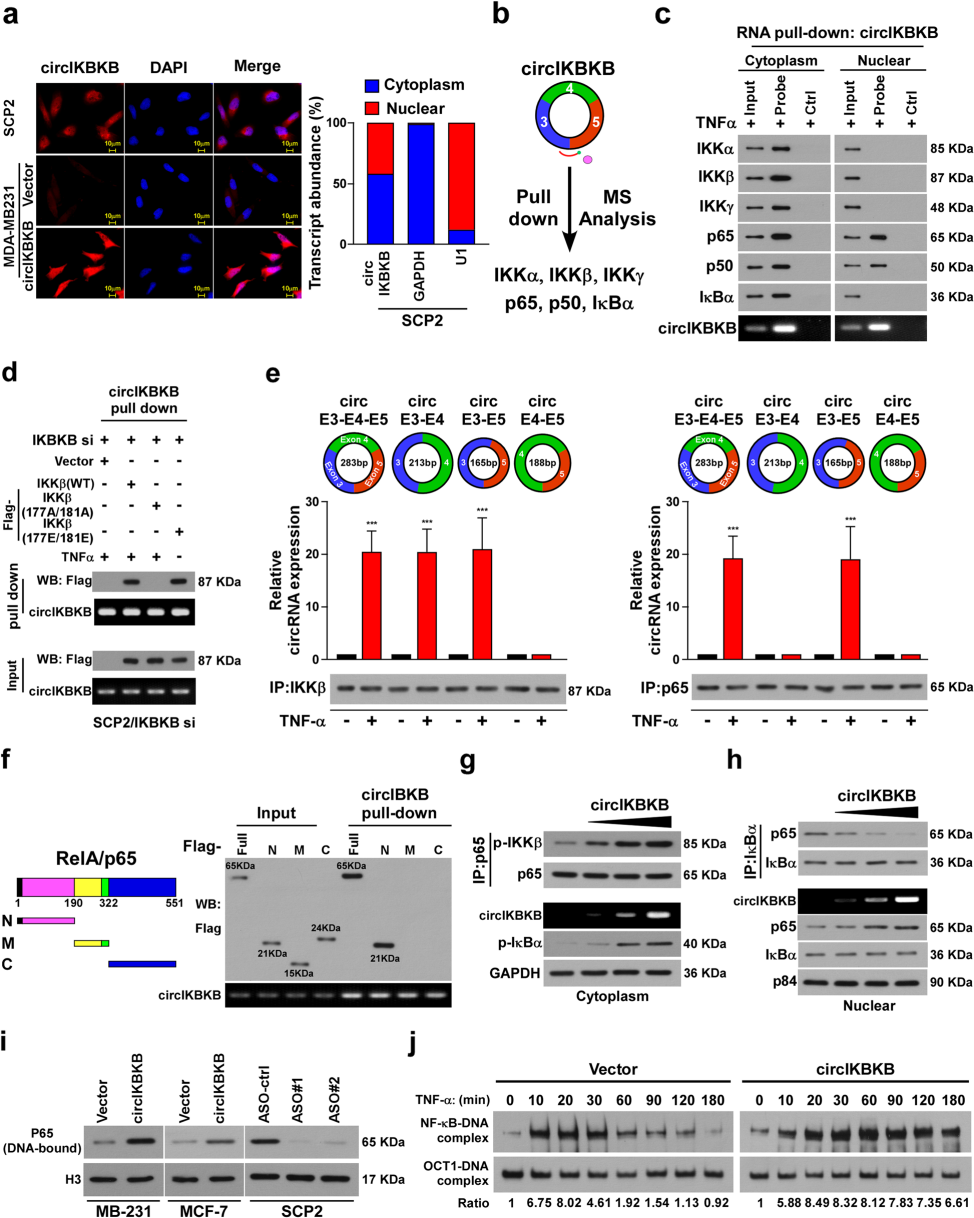
Cytoplasmic circIKBKB facilitates IKKβ-mediated IκBα phosphorylation and nuclear circIKBKB inhibited IκBα feedback loop. **a** FISH (left) and qPCR from nuclear-cytoplasmic fractionation analysis of subcellular localization of circIKBKB. **b** circIKBKB pull-down following the mass spectrometry showed that circIKBKB interacted with IKKα/IKKβ/IKKγ and p65/p50/IκBα complex. **c** RNA pull-down assay analysis of cytoplasmic and nuclear circIKBKB-interacting proteins (“probe” stands for circIKBKB probe, “Ctrl” stands for control probe). **d** RNA pull-down assay showing that circIKBKB only bound to active IKKβ. **e** RIP assay analysis of the binding region of circIKBKB with IKKβ or p65. **f** Schematic illustration of Flag-tagged full-length p65 and three truncated p65 fragments (left) and RNA pull-down assay analysis(right) of interaction between circIKBKB with p65 fragments. **g** IP (upper) and WB (lower) analysis showing that cytoplasm circIKBKB facilitated IKKβ/p65 interaction and IKKβ-mediated IκBα phosphorylation in a dose dependent manner. GAPDH served as loading control. **h** IP (upper) and WB (lower) analysis showing that nuclear circIKBKB inhibited IκBα feedback loop. P84 served as loading control. **i** Chromatin fraction and WB analysis of DNA-bound NF-κB in the indicated cells. H3 served as a loading control. **j** EMSA analysis of NF-κB activity in the indicated MDA-MB-231 cells treated with TNF-α (10 ng/ml) for the indicated times. Each error bar represents the mean ± SD of three independent experiments. * *P* < 0.05, ** *P* < 0.01, *** *P* < 0.001

To investigate the mechanism underlying circIKBKB-mediated NF-κB activation, we first examined the circIKBKB localization using fluorescence in situ hybridization (FISH) and subcellular fractionation assays. As shown in Fig. 4a, circIKBKB was distributed in both cytoplasm and nucleus. Interestingly, RNA pull-down assay following mass spectrometry (MS) showed that circIKBKB interacted with multiple key components in NF-κB signaling, including IKKα, IKKβ, IKKγ, p65, p50 and IκBα (Fig. 4b). However, RNA pull-down assays revealed that cytoplasmic circIKBKB could interact with both IKKα/IKKβ/IKKγ and p65/p50/IκBα complexes, whereas nuclear circIKBKB only interacted with p65/p50 complex (Fig. 4c), suggesting that circIKBKB might regulate NF-κB signaling in both cytoplasm and nuclear. Furthermore, in vitro binding assays showed that circIKBKB directly interacted with recombinant p65 and active IKKβ, but not with other recombinant proteins, suggesting that circIKBKB activated the NF-κB pathway through direct interaction with p-IKKβ and p65 (Fig. S5a). Consistent with this hypothesis, silencing IKKβ abrogated the interaction of circIKBKB with IKKα and IKKγ and silencing p65 abolished the association of circIKBKB with p50 and IκBα (Fig. S5b). RNA pull-down assays further demonstrated that circIKBKB interacted with constitutively active IKKβ (177E/181E) even without TNFα treatment but not with the kinase‐deficient IKKβ (177A/181A) mutant in TNFα-treated cells (Fig. 4d).

Moreover, we found that p-IKKβ could form complex with circRNA formed by IKBKB exon 3/4 or exon 3/5, but not with exon 4/5, indicating that exon 3 of circIKBKB was the binding region with IKKβ (Fig. 4e). However, deletion of either exon 3 or 5 abolished the circIKBKB/p65 interaction. These results suggest that the exon 3–5 back-splice junction is necessary for the circIKBKB/p65 complex formation (Fig. 4e).

### CircIKBKB sustains NF-κB signaling via inhibition of IκBα negative feedback

Since RNA pull-down assays showed that the N-terminal DNA-binding domain of p65, which is the IκBα-binding region, was the region that bound to circIKBKB (Fig. 4f). We speculated that circIKBKB and IκBα might competitively interact with NF-κB. Consistent with previous reports that IκBα forms a complex with NF-κB that removes NF-κB from DNA and translocates NF-κB back to the cytoplasm [25–27], overexpressing circIKBKB decreased the p65/IκBα complex formation, increased the level of DNA-bound NF-κB and persisted the TNF-α-induced nuclear accumulation of NF-κB. The opposite effects were observed in circIKBKB-silenced cells (Fig. 4g-i, Fig. S5c, d). Moreover, electrophoretic mobility shift assay (EMSA) indicated that the endogenous NF-κB DNA-binding activity after TNF-α treatment was significantly prolonged in circIKBKB-transduced cells compared to vector-control cells (Fig. 4j). Collectively, our results demonstrate that circIKBKB overexpression sustains NF-κB activity in BC cells.

### CircIKBKB recruits NF-κB to the promoters of multiple bone remodeling factors

**Fig. 5:**
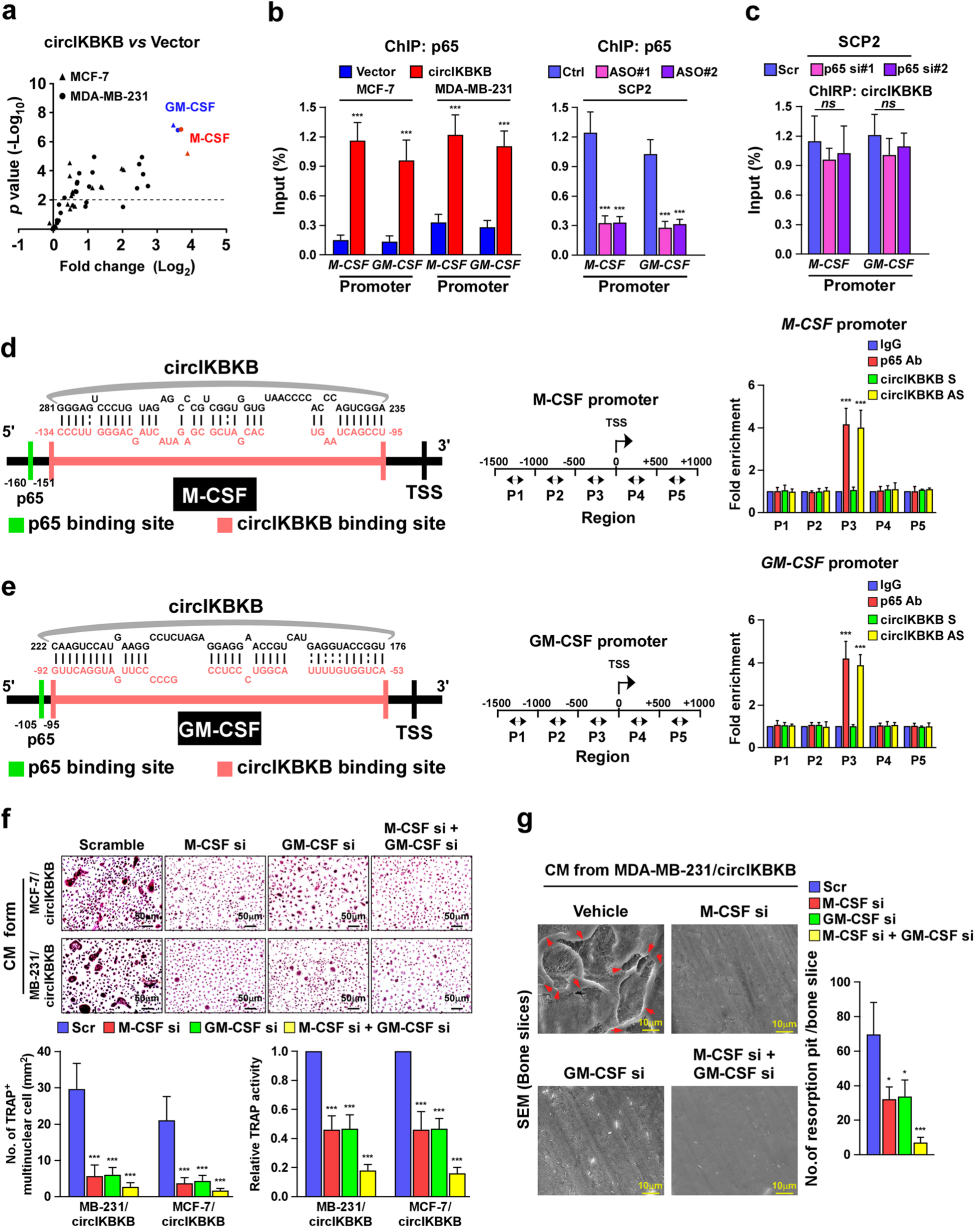
CircIKBKB directly interacts with recruits NF-κB to the promoters of multiple bone remodeling factors. **a** Real-time PCR analysis of mRNA levels of 25 bone-remodeling factor in the circIKBKB-overexpressing and control cells. GAPDH serve as a loading control. **b** ChIP assay analysis of the enrichment of p65 on the promoter of M-CSF and GM-CSF in the indicated cells. **c** ChIRP assay analysis of enrichment of circIKBKB on the promoter of M-CSF and GM-CSF in the p65-silenced and control cells. **d** Left and middle: Schematic illustration of the binding sites of circIKBKB and p65 on M-CSF promoter and PCR-amplified fragments of the M-CSF promoter. Right: ChIP and ChIRP assays analysis of enrichment of p65 and circIKBKB on the M-CSF promoter, respectively. **e** Left and middle: Schematic illustration of binding site of circIKBKB and p65 on GM-CSF promoter and PCR-amplified fragments of M-CSF promoter. Right: ChIP and ChIRP assays analysis of the enrichment of p65 and circIKBKB on the GM-CSF promoter, respectively. **f** Upper: Osteoclast differentiation assay by TRAP staining in the presence of CM from the indicated cells. Lower: Quantification of the number of TRAP^+^-multinuclear osteoclasts and TRAP activity from experiment in upper panel. **g** SEM images (left) and quantification of the number of resorption pits (right) of bone slices resorbed by pre-osteoclasts treated with CM from indicated cells. Each error bar represents the mean ± SD of three independent experiments. * *P* < 0.05, ** *P* < 0.01, *** *P* < 0.001

Next, we sought to identify the potential factors involved in circIKBKB-induced osteoclastogenesis and bone metastasis. qPCR analysis revealed that among total 25 bone remodeling factors, the mRNA levels of M-CSF and GM-CSF, which are also downstream targets of NF-κB, were significantly increased in circIKBKB-overexpressing BC cells but decreased in circIKBKB-silenced cells, compared to control cells, respectively (Fig. 5a and Fig. S6a). Concordantly, the secreted protein levels of M-CSF and GM-CSF were elevated in the CM from BC/circIKBKB cells and reduced in the CM from circIKBKB-silenced cells (Fig. S6b, c). Importantly, blocking NF-κB via overexpressing IκBα-mu drastically inhibited the induced effects of circIKBKB on M-CSF and GM-CSF expression (Fig. S6c, d). These results further support the crucial role of NF-κB in circIKBKB-induced osteoclastogenesis and bone metastasis.

Although overexpressing circIKBKB increased and silencing circIKBKB reduced the enrichment of NF-κB/p65 on M-CSF and GM-CSF promoters, ablating p65 did not change the level of circIKBKB on M-CSF and GM-CSF promoter (Fig. 5b, c). As we already demonstrated that circIKBKB was associated with NF-κB in the nuclear (Fig. 4c). Therefore, we hypothesized that circIKBKB might bind and recruit NF-κB to M-CSF and GM-CSF promoters. Analysis using the JASPAR (http://jaspar.genereg.net) and RNAInter (http://www.rna-society.org/rnainter/IntaRNA.html) platforms showed that the binding sites of circIKBKB and NF-κB on either M-CSF promoter or GM-CSF promoter are very close to each other and near to transactional start site (TSS), which was further confirmed by ChIP and ChIRP assays (Fig. 5d, e). Furthermore, we found that either using RNA interference (RNAi)-mediated silencing of M-CSF or GM-CSF or treatment with neutralizing anti-M-CSF or anti-GM-CSF antibody significantly abolished the inductive effect of circIKBKB on osteoclastogenesis and the bone-resorbing activity of osteoclasts (Fig. 5f, g and Fig. S6e, f). Therefore, these results indicate that circIKBKB recruits NF-κB to upregulates multiple bone remodeling factors, consequently inducing the formation of bone-metastatic environment.

### EIF4A3 increases circIKBKB expression via direct promotion of circIKBKB cyclization

**Fig. 6:**
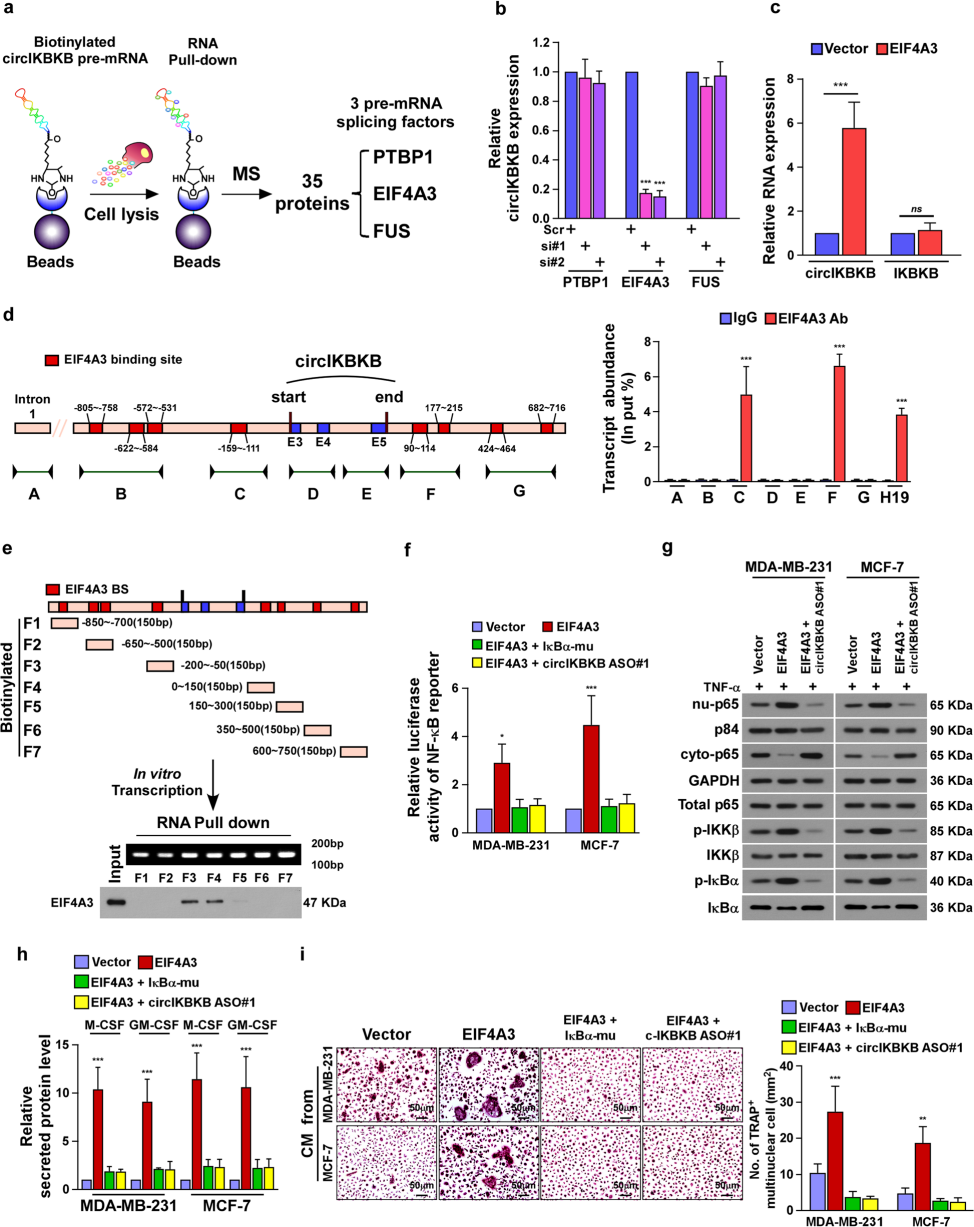
EIF4A3 increases circIKBKB expression via direct promotion of circIKBKB cyclization. **a** Schematic illustration of circIKBKB pre-mRNA pull-down following the mass spectrometry to identify circIKBKB cyclization factor. **b** Real-time PCR analysis of circIKBKB expression in the indicated SCP2 cells. GAPDH served as a loading control. **c** Real-time PCR analysis of circIKBKB and IKBKB expression in EIF4A3-overexpressing and control MDA-MB-231 cells. GAPDH served as a loading control. **d** The putative binding sites of EIF4A3 in the upstream and downstream region of the circIKBKB pre-mRNA predicted with circinteractome database (left), and RIP assay analysis of interaction of EIF4A3 with circIKBKB pre-mRNA in SCP2 cells. The IKBKB intron 1 was used as the negative control and H19 lncRNA was used as the positive control. **e** A schematic diagram of 7 fragments of circIKBKB pre-mRNA (upper) and RNA pull-down assay analysis (Lower) of the interaction between eIF4A3 and above 7 fragments of circIKBKB pre-mRNA. **f** Relative NF-κB-driven luciferase activity was analyzed in the indicated cells treated with TNF-α (2 ng/ml). **g** WB analysis of the level of cytoplasmic-p65, nuclear-p65, total p65, p-IKK-β, total IKK-β, p-IκB-α, and total IκB-α in the indicated cells treated with TNF-α (2 ng/ml). GAPDH served as a cytoplasmic control and p84 served as a nuclear control. **h** ELISA analysis of expression of secreted M-CSF and GM-CSF in CM from the indicated cells. **i** Image (left) and quantification (right) of TRAP^+^-multinuclear osteoclasts stimulated by CM from the indicated cells. Each error bar represents the mean ± SD of three independent experiments. * *P* < 0.05, ** *P* < 0.01, *** *P* < 0.001

To identify the potential factor(s) involved in circIKBKB cyclization, an RNA pull-down assay was performed using circIKBKB pre-mRNA, which was prepared via in vitro transcription, and followed the mass spectrometry-based proteomics analysis (Fig. 6a). All together 35 proteins were identified to be potent circIKBKB pre-mRNA-interacting proteins, including 3 pre-mRNA splicing factors, which were PTBP1, EIF4A3 and FUS (Fig. 6a). Further qRT-PCR analyses revealed that silencing EIF4A3 significantly reduced, but overexpressing EIF4A3 increased, the circIKBKB expression in BC cells (Fig. S7a, b and Fig. 6b, c). Moreover, overexpressing EIF4A3 did not affect the expression level of IKBKB (Fig. 6c), suggesting that EIF4A3 might be involved in circIKBKB cyclization.

We then examined whether EIF4A3 directly interacted with circIKBKB pre-mRNA. Circinteractome (https://circinteractome.nia.nih.gov/index.html) analysis showed 8 putative binding sites of EIF4A3 in the upstream and downstream regions of circIKBKB pre-mRNA (Fig. 6d). RNA immunoprecipitation (RIP) assays indicated that EIF4A3 was only associated with the putative binding sites near to exon 3 and exon 5 in circIKBKB pre-mRNA (Fig. 6d). These results were confirmed by RNA pull-down assays using in vitro transcript RNA fragments of the circIKBKB pre-mRNA (Fig. 6e). Therefore, these results demonstrate that EIF4A3 directly binds to the circIKBKB pre-mRNA and induces circIKBKB cyclization.

### EIF4A3 promotes osteoclastogenesis through circIKBKB/NF-κB signaling

Consistent with the inductive effect of EIF4A3 on circIKBKB expression as showed in above, overexpressing EIF4A3 significantly activated NF-κB signaling, as showed by increased NF-κB-luciferase activity and nuclear NF-κB level, and enhanced capability of BC cell to induce osteoclastogenesis, as indicated by elevated M-CSF and GM-CSF expression and the number of TRAP^+^-cells (Fig. 6f-i and Fig. S7c, d). However, the promotive effect of EIF4A3 on NF-κB signaling and osteoclastogenesis was dramatically abolished by either overexpressing IκBα-mu or silencing circIKBKB (Fig. 6f-i and Fig. S7c, d). Taken together, these results indicate that overexpression EIF4A3 promotes osteoclastogenesis through circIKBKB/NF-κB signaling.

### EIF4A3 overexpression correlates with breast cancer bone-metastasis

**Fig. 7:**
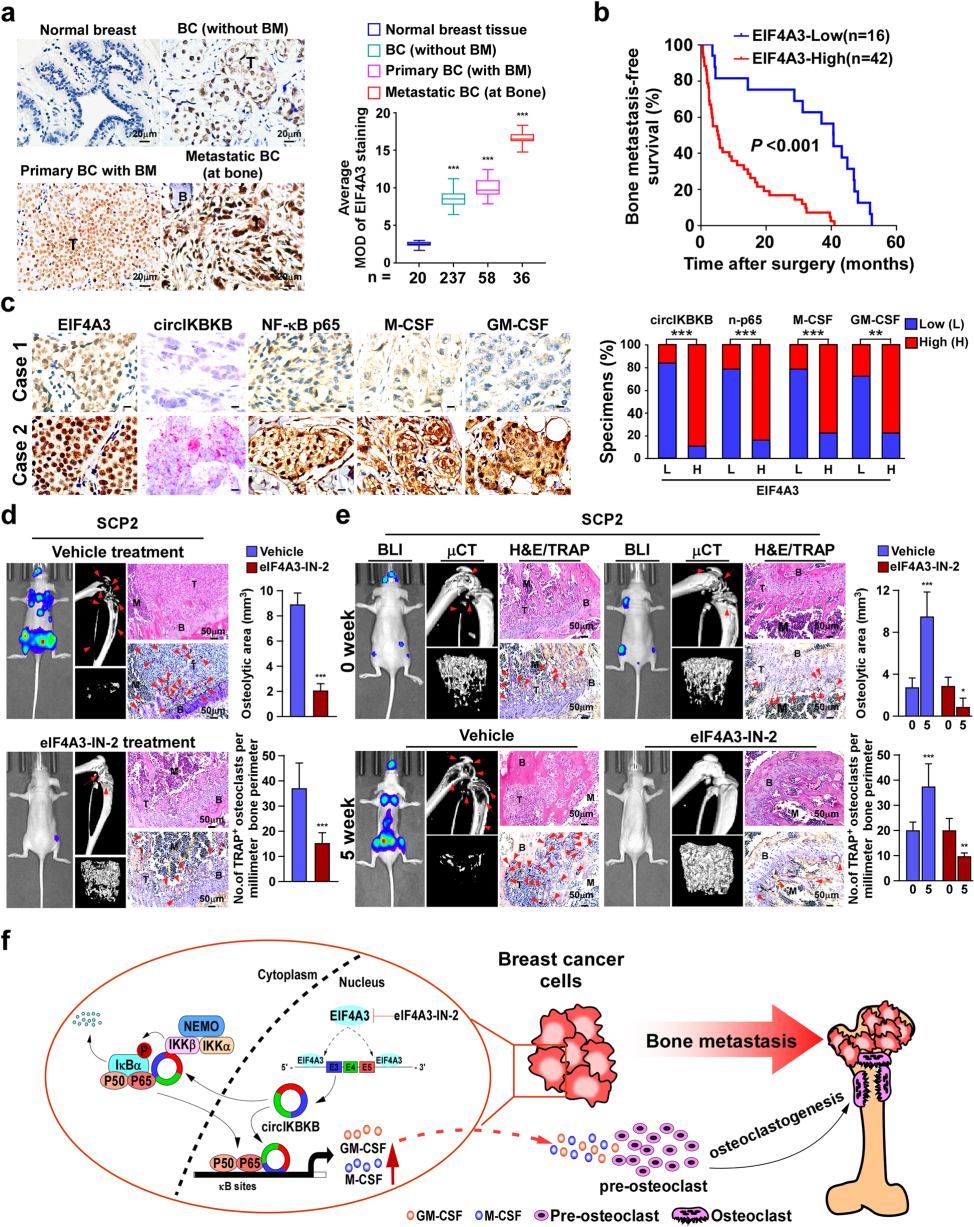
Pharmaceutical inhibition of eIF4A3 blocks bone-metastasis of BC cells in vivo. **a** IHC analysis (left) and quantification (right) of EIF4A3 expression in 20 normal breast tissues and 331 clinical BC tissues, including 295 primary BC tissues (237 non-BM/BC and 58 BM/BC) and 36 bone-metastatic BC tissues (at bone). **b** Kaplan–Meier analysis of bone metastasis-free survival curves in patients with BM/BC with low vs high expression of EIF4A3 (*n* = 58; *P* < 0.001, log-rank test). **c** Left: Two representative specimens are shown. Scale bars, 10 μm. Right: Percentages of specimens showing low or high EIF4A3 expression in relative to the levels of circIKBKB, nuclear-p65, M-CSF and GM-CSF. **d** Left and middle: BLI and μCT images (left) and histological (H&E and TRAP) images (middle) of bone lesions from mice, which received vehicle or eIF4A3-IN-2 treatment started at 2 days after intracardial injection of SCP2 cells. Right: Quantification of osteolytic sites and TRAP^+^ osteoclasts along the bone-tumor interface of metastases from experiment in left and middle panel. Scale bar, 50 μm. **e** Left and middle: BLI and μCT images (left) and histological (H&E and TRAP) images (middle) of bone lesions from mice, which vehicle or eIF4A3-IN-2 was started when bone-metastatic tumors formed. Right: quantification of osteolytic sites and TRAP^+^ osteoclasts along the bone-tumor interface of metastases from experiment in left and middle panel. **f** Model: EIF4A3-mediated circIKBKB overexpression activated NF-κB/bone remodeling factors signaling, resulting in inducing formation of bone pre-metastatic niche and breast cancer metastasis, and treatment with EIF4A3 inhibitor eIF4A3-IN-2 might serve as a promising approach to inhibit BC bone-metastasis. Each error bar represents the mean ± SD of three independent experiments. * *P* < 0.05, ** *P* < 0.01, *** *P* < 0.001

To further confirm whether EIF4A3 contributed to BC bone-metastasis clinically, IHC staining was performed in 20 normal breast tissues and 331 clinical BC tissues, including 295 primary BC tissues (237 without BM and 58 with BM) and 36 bone-metastatic BC tissues (at bone). As shown in Fig. 7a, compared to normal breast tissues, EIF4A3 expression was moderately increased in BC tissues (without BM) and primary BC tissues (with BM) but strongly elevated in bone-metastatic BC tissues (at bone). Importantly, patients with high EIF4A3-expressed BC had significantly shorter bone-metastasis-free survival than those with low EIF4A3-expressed BC (*P* < 0.001; Fig. 7b). These results suggest that EIF4A3 overexpression is clinically associated with BC bone-metastasis.

Moreover, we examined whether the abovementioned EIF4A3/circIKBKB/NF-κB axis identified in the in vitro and in vivo studies was clinically relevant in bone-metastatic BC tissues. The expression of circIKBKB was examined by *ISH* assay and the expression of NF-κB p65, M-CSF and GM-CSF were examined by IHC assay in 36 bone-metastatic BC tissues. Statistical analysis revealed that the EIF4A3 level was strongly correlated with the expression of circIKBKB (*P* < 0.001), nuclear p65 (*P* < 0.001), M-CSF (*P* < 0.001) and GM-CSF (*P* = 0.003). These data provided clinical evidence that the EIF4A3/circIKBKB/NF-κB axis-mediated upregulation of M-CSF and GM-CSF induced bone pre-metastatic niche formation, and consequently resulted in bone metastasis and poorer clinical outcomes in human breast cancer (Fig. 7c).

### Blocking EIF4A3 inhibits circIKBKB/NF-κB signaling and suppresses osteoclastogenesis

Consistent with the biological role of EIF4A3 in activation of circIKBKB/NF-κB signaling and the induction of osteoclastogenesis as shown in above, downregulation of EIF4A3 expression via RNAi technology or inhibition of EIF4A3 activity via treatment with eIF4A3-IN-2, a highly selective and noncompetitive EIF4A3 inhibitor, reduced circIKBKB expression and NF-κB-driven luciferase activity, and decreased M-CSF and GM-CSF in both transcriptional and translational levels (Fig. S8a-d). Meanwhile, we also observed that EIF4A3 inhibition also significantly decreased the capability of BC cells to induce osteoclastogenesis, as indicated by reduction the number of TRAP^+^-multinuclear osteoclasts and TRAP activity and lessened bone-resorbing activity (Fig. S8e).

### Pharmaceutical inhibition of EIF4A3 blocks bone-metastasis of BC cells in vivo

Finally, the in vivo effect of inhibition of EIF4A3 via eIF4A3-IN-2 treatment on BC-BM was examined. We first tested whether eIF4A3-IN-2 treatment could inhibit BC-BM, which eIF4A3-IN-2 treatment was started at 2 days after intracardial injection of SCP2 cells. As shown in Fig. S8f and g, eIF4A3-IN-2 treatment significantly delayed bone metastasis and reduced the onsets of bone-metastases and bone-metastasis burden compared to vehicle treatment. The eIF4A3-IN-2-treated mice displayed less osteolytic areas and number of TRAP^+^-osteoclasts in bone surface area (Fig. 7d). These results suggested that eIF4A3-IN-2 treatment might prevent BC-BM. Furthermore, we examined the therapeutic effect of eIF4A3-IN-2 on BC-BM, which eIF4A3-IN-2 treatment was started when bioluminescence signal of bone-metastatic tumors reached 2 × 10^5^ p/sec/cm^2^/sr (Fig. 7e). After 5 weeks treatment, the vehicle-treated mice exhibited rapid progress of bone metastasis, which showed more bone metastases and larger bone-metastatic tumor burden, accompanying with severe osteolytic bone lesions and higher numbers of TRAP^+^-osteoclasts along the bone-tumor interface. Strikingly, eIF4A3-IN-2 treatment dramatically reduced the onsets of bone metastases and decreased bone-metastatic tumor burden compared to vehicle treatment (Fig. 7e). Therefore, our results demonstrate that pharmaceutical inhibition of EIF4A3 not only prevents the initiation of BC-BM but also suppresses the progression of BC to bone-metastasis.

## Discussion

The overall survival of patients with BC has been improving significantly for the past two decades due to the marked progress in diagnostic techniques, multidisciplinary treatments, and implementation of surveillance programs. However, this concurrently provided time for tumor progression within the extramammary organs and the formation of metastatic foci at distant sites. The most common site of metastasis is the bone, which occurs in 65–80% of patients with metastatic breast cancer. Bone metastases not only dramatically reduces life expectancy to just 2–3 years following diagnosis but also severely affects the quality of life of patients by inducing skeletal-related events (SREs). However, bone metastasis of BC appears incurable clinically and the 5-year survival rates for these patients are below 25%. Currently, the main therapeutic interventions are focusing on disruption of the osteolytic cycle by targeting osteoclasts to extenuate and prevent SREs. Bisphosphonates and denosumab have been used to treat patients with BC-BM and could delay of the onset of SRES in patients [28] but also cause severe side effects, such as osteonecrosis of the jaw and hypercalcemia [29, 30]. Therefore, the development of novel therapeutic strategies for treating patients with BC-BM is urgently required. Herein, we reported that blocking EIF4A3 by eIF4A3-IN-2 significantly decreased circIKBKB expression and effectively prevented the initiation of BC-BM and also suppressed the progression of BC-BM. Therefore, our results might represent a new strategy for the treatment of BC-BM.

Breast cancer cells preferentially metastasize to specific organs, known as “organotropic metastasis”. The formation of primary tumors-induced microenvironment in distant organs/tissue sites, named pre-metastatic niche, has been demonstrated to be critical for subsequent engraftment and survival of metastatic cells, even determining metastatic organotropism [31, 32]. Consistently, tumor-produced cytokines have been proven to promote osteoclast-mediated bone resorption directly or indirectly, resulting in the release of bone matrix-stored growth factors, which further expanding tumors and generating a “vicious cycle” that supports bone metastasis [33, 34]. These studies suggest that identification of the specific factors that induce the formation of the bone pre-metastatic niche would be helpful for determining metastatic development before bone metastasis formation. In this study, we found that expression of GM-CSF and M-CSF, among 25 previous reported bone remodeling factors, was most significantly increased in conditioned media from circIKBKB-overexpressing breast cancer cells. Importantly, treatment with neutralizing anti-M-CSF or anti-GM-CSF antibodies significantly abolished the inductive effect of circIKBKB on osteoclastogenesis and the bone-resorbing activity of osteoclasts. These results indicated that GM-CSF or M-CSF were the key factors in conditioned media to exert on osteoclastogenesis. Consistent with our findings, Park BK, et al. reported that GM-CSF was a key factor to promote osteolytic bone metastasis of breast cancer by stimulating osteoclast development [18]. Meanwhile, it has been also reported that M-CSF protein secreted from breast cancer cells also contributed to osteoclast formation and repression of M-CSF suppressed osteoclastogenesis and tumor-induced osteolysis in an orthotopic breast cancer bone metastasis mouse model [17, 35]. We further demonstrated that circIKBKB could directly associate with the promoters of M-CSF and GM-CSF and upregulate M-CSF and GM-CSF levels. Thus, our results unveiled a decisive role of circIKBKB in promotion of bone metastasis by inducing pre-metastatic niche formation and suggested that circIKBKB might be a useful biomarker for evaluating bone metastasis in BC.

Interestingly, as downstream target of transcriptional factor NF-κB [36, 37], either M-CSF or GM-CSF could also positively feedback to activate NF-κB signaling pathway [38, 39], which suggested that M-CSF and GM-CSF might regulate each other. Indeed, two previous studies have reported that GM-CSF treatment could upregulate endogenous M-CSF in human monocytes [40, 41]. Herein, we found that overexpressing circIKBKB significant activated NF-κB pathway, resulting in significant upregulation of M-CSF and GM-CSF, and silencing either M-CSF or GM-CSF or treatment with neutralizing anti-M-CSF or anti-GM-CSF antibody significantly abolished the inductive effect of circIKBKB on osteoclastogenesis and the bone-resorbing activity of osteoclasts. Therefore, we speculated that overexpressing induced M-CSF and GM-CSF expression via activation of NF-κB signaling, and circIKBKB-induced M-CSF and GM-CSF might feeded back to further strengthen the circIKBKB-mediated NF-κB activation, which led to osteoclastogenesis and breast cancer bone metastasis.

Constitutive overactivation of NF-κB signaling has been found in multiple cancer types, including BC. Functioning as natural NF-κB inhibitor, IκBα is also one of the transcriptional targets of NF-κB. The activated NF-κB-mediated resynthesis of IκBα removes NF-κB from chromatin and exports NF-κB back to the cytoplasm, which forms feedback loop to prevent constitutive NF-κB overactivation [25–27]. However, how BC cells override the NF-κB/IκB negative feedback loop, which maintains the hyperactivated NF-κB, remains unclear. In the current study, we found that circIKBKB interacted with the NF-κB N-terminal, which is the IκBα-binding region. Overexpression of circIKBKB decreased the p65/IκBα complex formation, persisted the TNF-α-induced nuclear accumulation of NF-κB and increased the level of DNA-bound NF-κB, indicated that circIKBKB competitively interacted with N-terminal of NF-κB with IκBα. Thus, our finding represents a novel mechanism in which circRNA mediated NF-κB hyperactivation through disrupting the NF-κB/IκBα negative feedback loop in BC. Considering that hyperactivation of NF-κB frequently found in many types of cancer, it is worthy to further investigate the expression and role of circIKBKB in other cancers.

## Conclusions

Disrupting the interaction between the circulating breast cancer cells (seeds) and bone microenvironment (soil), which form a vicious cycle to support metastatic cancer cells proliferationc and survival in the bone tissue, will be beneficial for a large group of patients with bone metastasis. Herein, we demonstrated that a novel circRNA circIKBKB played a vital role in inducing bone pre-metastatic niche formation via sustaining NF-κB/bone remodeling factors signaling, consequently leading to breast cancer bone metastasis. Considering the high stability and abundance of circRNA, further investigation of the expression and role of circIKBKB in other cancers will not only provide valuable insights to better understand the mechanism driving the pre-metastatic niche formation but also may develop novel therapeutic strategies for treatment of human cancers.

## Supplementary Information

**Additional file 1: Table S1.** The original sequencing results of all differentially expressed circRNAs in six breast cancer (without BM) and six breast cancer (with BM) tissues.

**Additional file 2: Table S2.** Primers, Probes, siRNA, shRNA and ASOs used in this study.

**Additional file 3: Table S3.** Pathways of the Cignal Finder 45‐Pathway Arrays.

**Additional file 4.** Supplementary Methods and Figures.

## Data Availability

The datasets used and/or analyzed during the current study are available within the manuscript and its supplementary information files.

## Abbreviations

BC: Breast cancer
circRNAs: Circular RNAs
BPs: Bisphosphonates
RANKL: Anti-receptor activator of nuclear factor kappa B ligand
BC-BM: BC-bone metastasis
NF-κB: Nuclear factor kappa B
PTHrP: Parathyroid hormone-related protein preproprotein
IL-6: Interleukin 6
M-CSF: Macrophage colony stimulating factor
GM-CSF: Granulocyte–macrophage colony stimulating factor
IKK: IκB kinase
IκBα: Nuclear factor kappa B inhibitor alpha
ROS: Reactive oxygen species
IKBKB: Inhibitor of nuclear factor kappa B kinase subunit beta
EIF4A3: Eukaryotic translation initiation factor 4A3
non-BM/BC: BC tissues without BM
BM/BC: Bone-metastatic BC tissues
RT-PCR: Reverse transcription PCR
qRT-PCR: Quantitative real-time PCR
ISH: In situ hybridization
ASO: Antisense oligonucleotide
BLI: Bioluminescence imaging
μCT: Micro- computed tomography
TRAP: Tartrate-resistant acid phosphatase
C-fos: FBJ osteosarcoma oncogene
Acp5: Acid phosphatase 5, tartrate resistant
Ctsk: Cathepsin K
Nfat-c1: Nuclear factor of activated T cells 1
Dc-stamp: Dendrocyte expressed seven transmembrane protein
pre-oc: Pre-osteoclasts
CM: Conditioned media
ALP: Alkaline phosphatase
OPG: Osteoprotegerin
IF: Immunofluorescence
TGF-β: Transforming growth factor beta
IκBα-mu: NF-kB inhibitor
IKK2-I VI: IKK-β inhibitor VI
FISH: Fluorescence in situ hybridization
MS: Mass spectrometry
EMSA: Electrophoretic mobility shift assay
TSS: Transactional start site
ChIP: Chromatin immunoprecipitation
RNAi: RNA interference
RIP: RNA Immunoprecipitation
SREs: Skeletal-related events
