# Supplementary material for: Circular RNA circIKBKB promotes breast cancer bone metastasis through sustaining NF-κB/bone remodeling factors signaling
Source: Mol Cancer. 2021 Jul 29;20:98. doi: 10.1186/s12943-021-01394-8 (PMC8320207; doi:10.1186/s12943-021-01394-8)
Supplement: Supplementary file 4 — Additional file 4. Supplementary Methods and Figures. [file 12943_2021_1394_MOESM4_ESM.docx]

**Circular RNA circIKBKB promotes breast cancer bone metastasis through sustaining**

**NF-κB/bone remodeling factors signaling**

Yingru Xu, Shuxia Zhang, Xinyi Liao, Man Li, Suwen Chen, Xincheng Li, Xingui Wu, Meisongzhu Yang, Miaoling Tang, Yameng Hu, Ziwen Li, Ruyuan Yu, Mudan Huang, Libing Song, Jun Li *

These authors contributed equally: Yingru Xu, Shuxia Zhang

**SUPPLEMENTARY METHODS AND FIGURES**

**INDEX**

**Supplementary Methods……..……………………………………………..……..….Page 2**

**Supplementary Figures.…………………………………………….…....…....…..….Page 11**

**Supplementary References.…………………………...………….….....…..………...Page 25**

**Supplementary Methods**

**Cell lines**

The MDA-MB-231, MCF-7 and SKBR3 cell lines were obtained from the Sun Yat-sen University Cancer Center. SCP2 and 4175 cell lines were a kind gift from Guohong Hu. All the cell lines were grown in Dulbecco’s modified Eagle’s medium (DMEM) (Gibco, Grand Island, NY) supplemented with 10% fetal bovine serum (Gibco, Grand Island, NY), according previously report[1, 2]. All the cell lines were tested for mycoplasma contamination and were authenticated by short tandem repeat (STR) fingerprinting at Medicine Lab of Forensic Medicine Department of Sun Yat-Sen University (China).

**Plasmids, retroviral infection and transfection**

The human IҡBα, IҡBα-mu and truncated-p65 fragments were cloned into pCDEF vector. ShRNAs targeting EIF4A3 and were cloned into the pSuper retro viral vector. All primers and oligonucleotides used in plasmid construction are listed in Table S2. Transfection of short interfering RNAs (siRNAs) or plasmids was performed using the Lipofectamine 3000 reagent (Thermo Fisher Scientific, Waltham, MA, USA) according to the manufacturer’s instructions. Stable cell lines expressing circIKBKB or EIF4A3 were generated via retroviral infection and selected for 10 days with 0.5 µg/ml puromycin 48 h after infection, according previously report[3].

**RNA extraction, reverse transcription and** **Quantitative Real-time PCR**

Total RNA was extracted from the indicated cells using the Trizol (Life Technologies, Carlsbad, CA, USA) reagent according to the manufacturer’s instruction. Quantitative Real-time reverse transcription-polymerase chain reaction (PCR) primers and probes were designed with the assistance of the Primer Express v 2.0 software (Applied BioSystems, Foster City, CA, USA). Expression data were normalized to the geometric mean of housekeeping gene GAPDH to control the variability in expression levels and calculated as 2^- [(Ct of gene) – (Ct of GAPDH)]^, where Ct represents the cycle threshold for each transcript. All primers are listed in Table S2.

**Chemical reagents**

Actinomycin D (Act D, S8964) was purchased from Selleck Chemicals (Houston, TX, USA). Human recombinant IKKα protein (PV4327 , Thermo Fisher Scientific, Waltham, MA, USA), recombinant active IKKα protein (ab102103; Cambridge, MA, USA), recombinant active IKKβ protein (ab177584; Cambridge, MA, USA), recombinant IKKβ protein (ab114243; Cambridge, MA, USA), recombinant IKKγ protein (ab125589; Cambridge, MA, USA), recombinant p65 protein (ab16636; Cambridge, MA, USA), recombinant p50 protein (ab112327; Cambridge, MA, USA) and recombinant IKBα protein (ab59981; Cambridge, MA, USA) were purchased from Abcam. RNase R (Cat#RNR07250; UW-Madison, USA) were purchased from Epicentre® (an Illumina company).

**RNase R/Actinomycin D**

Total RNA (2 μg) was incubated with 3 U/μg RNase R (Epicentre Technologies, Madison, WI, USA) for 15 min at 37 °C. BC cells were transferred to six-well plates and treated with 5 μg/ml actinomycin D when the number of cells reached 9×10^5^, with samples collected at the indicated time points. The expression of circIKBKB and the linear counterpart mRNA IKBKB was analysed by qRT-PCR.

**Luciferase assay**

According previously report[4], the cells (1 × 10^3^) were seeded in triplicate in 48-well plates and allowed to settle for 24 h. One hundred nanograms of luciferase reporter plasmids or the control-luciferase plasmid, plus 5 ng of pRL-TK renilla plasmid (Promega, Madison, WI), were transfected into indicated cells using the Lipofectamine 3000 reagent (Invitrogen, Carlsbad, CA, USA) according to the manufacturer’s recommendation. Luciferase and renilla signals were measured at 48 h after transfection using the Dual Luciferase Reporter Assay Kit (Promega, Madison, WI) according to a protocol provided by the manufacturer.

**RNA fluorescence *in situ* hybridization**

Cy3-labelled circIKBKB probes were designed and synthesized by RiboBio. A fluorescence *in situ* hybridization (FISH) kit (RiboBio) was used to detect the probe signals in BC cells according to the manufacturer’s instructions. Nuclei were stained with 4,6-diamidino-2-phenylindole (DAPI). All images were captured using the AxioVision Rel.4.6 computerized image analysis system (Carl Zeiss, Jena, Germany).

**Immunohistochemistry (IHC)**

IHC was performed on formalin-fixed, paraffin-embedded human tissue sections as described before[5]. IHC analysis was performed to determine altered protein expression in paraffin-embedded normal breast tissues, breast tumor tissues and bone metastasis tissues with anti-EIF4A3 (Abcam, ab180573), anti-p65 (Abcam, ab32536), anti-M-CSF (Abcam, ab233387), and anti-GM-CSF (Proteintech, 17762-1-AP) antibodies overnight at 4℃. The degree of immunostaining of formalin-fixed, paraffin-embedded sections were reviewed and scored separately by two independent pathologists blinded to the histopathological features and patient data of the samples. The scores were determined by combining the proportion of positively-stained tumor cells and the intensity of staining. The scores given by the two independent pathologists were combined into a mean score for further comparative evaluation. Tumor cell proportions were scored as follows: 0, no positive tumor cells; 1, <10% positive tumor cells; 2, 10%–35% positive tumor cells; 3, 35%–75% positive tumor cells; 4, >75% positive tumor cells. Staining intensity was graded according to the following standard: 1, no staining; 2, weak staining (light yellow); 3, moderate staining (yellow brown); 4, strong staining (brown). The staining index (SI) was calculated as the product of the staining intensity score and the proportion of positive tumor cells. Using this method of assessment, we evaluated protein expression in normal breast tissues, breast tumor tissues and bone metastasis tissues by determining the SI, with possible scores of 0, 2, 3, 4, 6, 8, 9, 12, and 16. Samples with a SI ≥ 8 were determined as high expression and samples with a SI < 8 were determined as low expression. Cutoff values were determined on the basis of a measure of heterogeneity using the log-rank test with respect to overall survival.

**Mean optical density (MOD) analysis**

ISH staining for RNA expression of circIKBKB and IHC staining for protein expression of EIF4A3, M-CSF and GM-CSF in BC samples with bone or no-bone metastasis was quantitatively analyzed by using the AxioVision 4.6 computerized image analysis system assisted with an automatic measurement program (Carl Zeiss). The method of mean optical density (MOD) was used to determine the immunostaining intensity of each tested specimen. Briefly, the stained sections were evaluated at ×200 magnification, and 10 representative staining fields of each section were analyzed to verify the MOD, which represents the strength of staining signals as measured per positive pixels, according previously report[6]. The MOD data were statistically analyzed by using the t test to compare the average MOD difference between different groups of tissues, and *P* < 0.05 was considered significant.

**Immunoblotting analysis (IB)**

IB was performed according to a standard protocol, according previously report[7], with the following antibodies: p65 (ab76302) antibodies, p-IKKβ (ab194528) antibodies, IKKβ (ab264239) antibodies, p-IKKα (ab17943) antibodies, IKKα (ab109749) antibodies, HA (ab137838) antibodies, p50 (ab32360) antibodies, Flag (ab1162) antibodies, IKBα (ab32518) antibodies, H3 (ab1791) antibodies, EIF4A3 (ab180573) antibodies, PTBP1 (ab180573) antibodies and FUS (ab124923) antibodies were purchased from Abcam (Cambridge, MA, USA). IKKγ (18474-1-AP) antibodies, P84 (10920-1-AP) antibodies, GAPDH (10494-1-AP) antibodies and α-Tubulin (11224-1-AP) antibodies purchased from Proteintech (Rosemont, USA). p-IKBα (#9246) antibodies purchased from Cell Signaling Technology (SHH, CHN).

**Immunofluorescence (IF) staining**

The osteoclast precursors, mouse bone marrow cells were placed in 24-well clusters containing glass coverslips (Thermo Fisher Scientific) for 24 h and were treated with conditioned media (CM) from BC cells for 6 days, according previously report[6]. Cells were rinsed briefly with PBS (pH7.4) and fixed in 4% (w/v) paraformaldehyde in PBS (pH7.4) for 20 min at 37 °C. Aspirate fixation solution and wash cells 2-3 times in PBS (pH7.4). And followed by the antibody: anti-p65 (Abcam, ab32536) antibody. The secondary antibody was goat anti-rabbit IgG (H + L) conjugated with Alexa Fluor 594 (Thermo Fisher Scientific), or added 1:1000 dilution of Phalloidin-iFluor 488 (ab176753) in 1% BSA at room temperature for 20-90 minutes. Rinse cells 2-3 times with PBS (pH7.4) (5 min/wash). Then cells were mounted with Antifade Mountant with DAPI (Thermo Fisher Scientific). Observe the cells at Ex/Em 493/517 nm and the images were captured using the AxioVision Rel.4.6 computerized image analysis system (Carl Zeiss, Jena, Germany).

**Enzyme-linked immunosorbent assay (ELISA)**

Serum samples were kept at room temperature for about 1.0 h to defrost completely before assays. The levels of mouse serum M-CSF in supernatants were measured using a mouse M-CSF enzyme-linked immunosorbent assay kits (Abcam, ab199084). The levels of mouse serum GM-CSF in supernatants were measured using a mouse GM-CSF enzyme-linked immunosorbent assay kits (Abcam, ab201276). The M-CSF level in the culture medium from BC cells were measured using a human M-CSF enzyme-linked immunosorbent assay kits (Abcam, ab245714). The GM-CSF level in the culture medium from BC cells were measured using a human GM-CSF enzyme-linked immunosorbent assay kits (Abcam, ab174448) and analyzed according to the manufacturer's instructions. Data were read with the SpectraMax i3x Multi-Mode Microplate Reader (Molecular Devices) at 450 nm, the concentrations of M-CSF or GM-CSF in the samples were determined by extrapolating from the standard curve created by plotting the absorbance of the standards versus corresponding concentrations.

**Chromatin Immunoprecipitation (ChIP) assay**

The entire procedure was performed with the chromatin immunoprecipitation (ChIPs) assay kit (Cell Signaling Technology, Danvers, MA, USA) according to the manufacturer's instructions. Briefly, indicated cells were grown to 70~80% confluence on 100-mm culture dish and were fixed with 1% formaldehyde to cross-link proteins to DNA. The cell lysates were sonicated to shear DNA into small uniform fragments. Equal aliquots of chromatin supernatants were then immunoprecipitated overnight at 4°C using anti-p65 (CST, #8242) and anti-IgG antibodies (Millipore, Billerica, MA) with protein G magnetic beads. The cross-linked protein/DNA complexes were collected by magnetic pull down, and then were eluted from beads by elution buffer. After reverse cross-link of protein/DNA complexes to free DNA, PCR was performed using specific primers. All ChIP primers are listed in Table S2.

**Osteoclastogenesis assay**

The mouse bone marrow cells (1 × 10^5^) were cultured on 24-well clusters containing glass coverslips (Thermo Fisher Scientific) and grown in the conditioned media (CM) from indicated BC cells, according previously report[6]. Media were changed every other day. Osteoclasts were counted on day 6. The osteoclasts cultured on plastic dishes were fixed with 4% paraformaldehyde/PBS (pH7.4), and TRAP expression was examined by staining with a commercial kit (387A-1KT; Sigma-Aldrich). Osteoclasts were defined as TRAP-positive multinucleated cells containing more than 3 nuclei.

**Bone resorption pit assay**

To study the ability of osteoclasts to form resorption pits on bone slices (IDS PLC, catalog number: DT-1BON1000-96), the mouse bone marrow cells (1 × 10^5^) were seeded onto the bone slices. Then cells were cultured for 9 days in conditioned media (CM) from BC cells with indicated treatment. Media were changed every other day. After 9 days, bone slices were fixed with 2.5% glutaraldehyde, followed by the removal of cells by mechanical agitation and sonication. Resorption lacunae were visualized by scanning electron microscopy (SEM). Three fields were randomly selected for each bone slice for further analysis. Pit areas were quantified using ImageJ software.

**Micro-CT (μCT) analysis**

The hind limbs were removed from euthanized mice and fixed in either 4% paraformaldehyde solution or periodate-lysine-paraformaldehyde fixative. Fixed hind limbs were dissected free of tissue and scanned with high-resolution images (spatial resolution, 15 micron) on a micro-CT scanner (SIEMENS, Munich, Germany) to measure osteolytic region in tibia and femur of hind limb, and the perimeters, including trabecular volume, trabecular number, trabecular thickness, trabecular separation and trabecular bone pattern factor, were statistically analyzed to determine the degree of osteolysis. The images were reconstructed using Inveon Research Workplace (IRW) software (Inveon v. 04.00). A standard trabecular volume of interest was chosen starting 0.1 mm from the growth plate and included all trabeculae in a 1 mm^3^ region of bone. Trabecular volume and number were assessed in this region. Osteolytic lesions were measured through 360^o^ view of the bone on a three-dimensional model in a 3 mm length of cortical bone, starting at the growth plate.

**In vivo quantification of osteoclast number**

Hind limbs were fixed in paraformaldehyde solution (4%), decalcified in 14.3% EDTA for 4 days at 37 ^o^C with daily changes of EDTA, then embedded in paraffin wax. Sections were used by H&E stained with Mayer’s hematoxylin solution or stained with TRAP (a TRAP kit, 387A-1KT; Sigma-Aldrich) according to manufacturer’s protocols. The numbers of TRAP+-osteoclasts were determined on a 3 mm length of endocortical surface and viewed on an optical microscope (Olympus, DP72, Tokyo, Japan).

**Alkaline phosphatase (ALP) staining**

Fourteen days after osteogenic induction, a TRAP/ALP Stain Kit (Wako, Richmond, VA, USA; 294-67001) was used to perform alkaline phosphatase (ALP) staining, according to the manufacturer's instructions. The cells cultured in 24-well plates were rinsed three times by PBS (pH7.4) and fixated for 30 min using pre-cold fixative. Next, ALP substrate solution was added and cells were cultivated (room temperature, 15-45min) in a darkroom. After that, cells with ddH_2_O were rinsed and filmed under an optical microscope. The absorbance at 405 nm of each well was measured with a microplate reader according to the manufacturer’s instruction.

**Preparation of nuclear and cytosol extracts**

Nuclear and cytosol fractions were isolated by using PARIS™ Kit (Thermo Fisher Scientific, Waltham, MA, AM1921) according to the manufacturer’s instructions. The indicated cells were treated with 0.25% trypsin-EDTA and subsequently harvested at centrifuge at 500 × g for 5 minutes. The cell pellet was washed 2-3 times with PBS (pH7.4) and then transferred to a 1.5mL microcentrifuge tube and centrifuged at 500 × g for 2-3 minutes. The cell pellet was resuspended in 300 μL ice-cold Cell Fraction Buffer and vortexed the tube vigorously and incubated on ice for 10 min, and centrifuged for 5 minutes at 500 × g. The supernatant (cytoplasmic extract) was immediately transfer to a clean pre-chilled tube. The pelleted nuclei were resuspended in 300 μL ice-cold Cell Disruption Buffer, which contains nuclei and vortexed for seconds every 10 minutes, for a total of 40 minutes. The supernatant (nuclear extract) fraction was then centrifuged at ~500 × g for 10 minutes and then immediately transferred to a clean pre-chilled tube. Store extracts at -80°C until use.

***In Situ* Hybridization (ISH)**

Tissue samples were fixed in neutral buffered formalin (NBF 10%) and paraffin-embedded (FFPE) according to standard protocols. ISH was performed on 4.5-μm thick sections on a Ventana Discovery XT automation system (Ventana Medical Systems, Tucson, AZ) using RNAscope® 2.5 HD Reagent Kit- Red (322350, Advanced Cell Diagnostics, Hayward, CA). Sections were hybridized with bacterial DapB (dihydrodipicolinate reductase), human PPIB (peptidylprolyl isomerase B) and nonoverlapping human circIKBKB probes. Slides were scanned into digital images on an Olympus VS120 (Olympus, Hamburg, Germany). The sequences of the probes are available in Table S2.

**Electrophoretic Mobility Shift Assay (EMSA)**

EMSA was performed by using the LightShift Chemiluminescent EMSA kit from Pierce Biotechnology (Rockford, IL) according to the manufacturer’s standard protocol. Following DNA probes containing speciﬁc binding sites were used: NF-κB: sense, 5’-AGTTGAGGGGACTTTCCCAGGC-3’, antisense, 5’-GCCTGGGAAAGTCCCCTCAAC-3’; OCT-1: sense, 5’-TGTCGAATGCA AATCACTAGAA-3’, antisense, 5’-TTCTAGTGATTTGCATTCGACA-3’

**Chromatin isolation by RNA purification (ChIRP)**

CircIKBKB antisense probe was designed at the back-spliced site. All probes were synthesized with BiotinTEG at the 3′ end. BC cells were harvested for ChIRP assay as previously described[8]. The sequences of the probes are available in Table S2.

**RNA pulldown assay**

The biotin-labeled circIKBKB or control probe was incubated with streptavidin magnetic beads at room temperature for 1 h. Then the BC cells lysates were incubated with probe-beads complex at 4 °C overnight for the binding of RNA-associated proteins to RNA. Subsequently, the RNA-protein complexes were washed three times and eluted from beads. The eluted proteins were finally analyzed by mass spectrometry or Western blot.

**RNA immunoprecipitation (RIP)**

A Magna RNA binding protein immunoprecipitation kit was purchased from Millipore, and the experiment was conducted according to the manufacturer’s instructions. In brief, a total of 5 × 10^7^ cells were harvested and lysed using RIP lysis buffer. RNA-binding protein was immunoprecipitated using an anti-EIF4A3 specific antibody (ab180573, Abcam, USA), and the retrieved RNA was subjected to qRT-PCR analysis. An anti-IgG antibody was used as a negative control. For the qRT-PCR analysis, circIKBKB pre-mRNA Intron 1 was used as a nonspecific control, and lncRNA H19 was used as a positive control.

**Cignal^TM^ finder pathway reporter assay**

To use the Cignal Finder 45‐Pathway Arrays (CCA 901L; Qiagen, Germany; Tables S3) in the plate format, a reverse transfection method must be employed. This approach involved seeding the tumor cell line onto the transfection complexes (containing the Attractene transfection reagent [Qiagen, Germany] and test nucleic acids) on the first day following the manufacturer's protocol. Then, the luciferase assay was carried out using the Dual‐Luciferase Reporter Assay System (Promega, USA) following the manufacturer's protocol for developing the assay.

**Supplementary Figures**


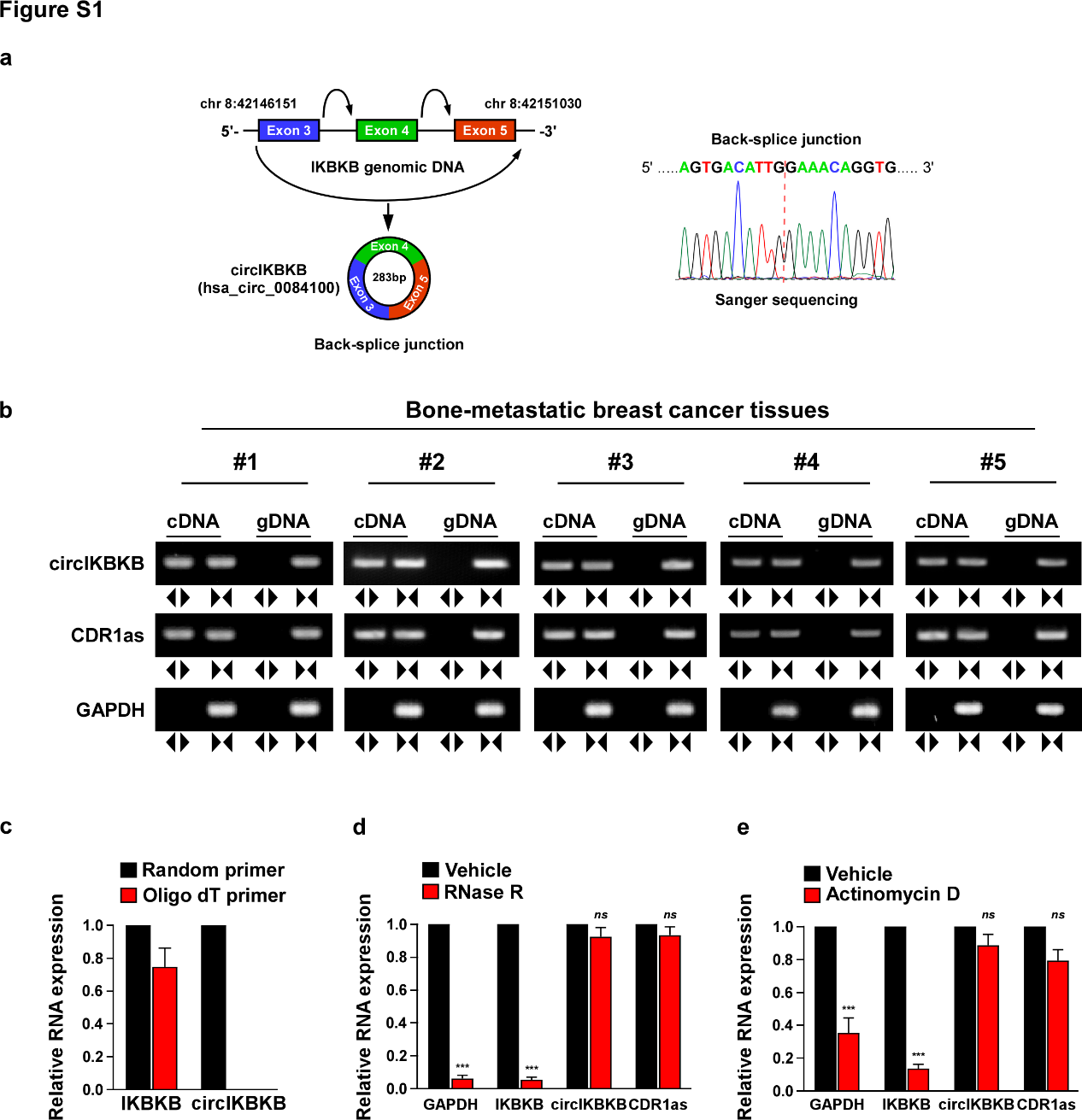


**Figure S1.** Silencing circIKBKB inhibits osteolytic bone metastasis of BC *in vivo*.  **a.** Schematic illustration of circIKBKB formation via the circularization of exons 3-4-5 in IKBKB gene. The back-splice junction sequence of circIKBKB was validated by Sanger sequencing. **b.** The existence of circIKBKB was confirmed by RT-PCR and gel electrophoresis in 5 bone-metastatic BC tissues using the divergent and convergent primers, respectively. Divergent primers amplified circIKBKB in cDNA but not genomic DNA (gDNA), along with CDR1as as a positive control, GAPDH as a negative control. **c**. qRT-PCR analysis of expression of circIKBKB and linear IKBKB using random hexamer or oligo(dT)_18_ primers in SCP2 cells. GAPDH served as a loading control. **d**. qRT-PCR analysis of stability of circIKBKB and linear IKBKB in the RNase R-treated SCP2 cells, along with CDR1as as a positive control, GAPDH as a negative control. **e**. qRT-PCR analysis of stability of circIKBKB and linear IKBKB in the Actinomycin D-treated SCP2 cells, along with CDR1as as a positive control, GAPDH as a negative control. Each error bar represents the mean ± SD of three independent experiments. * *P* < 0.05, ** *P* < 0.01, *** *P* < 0.001.

**
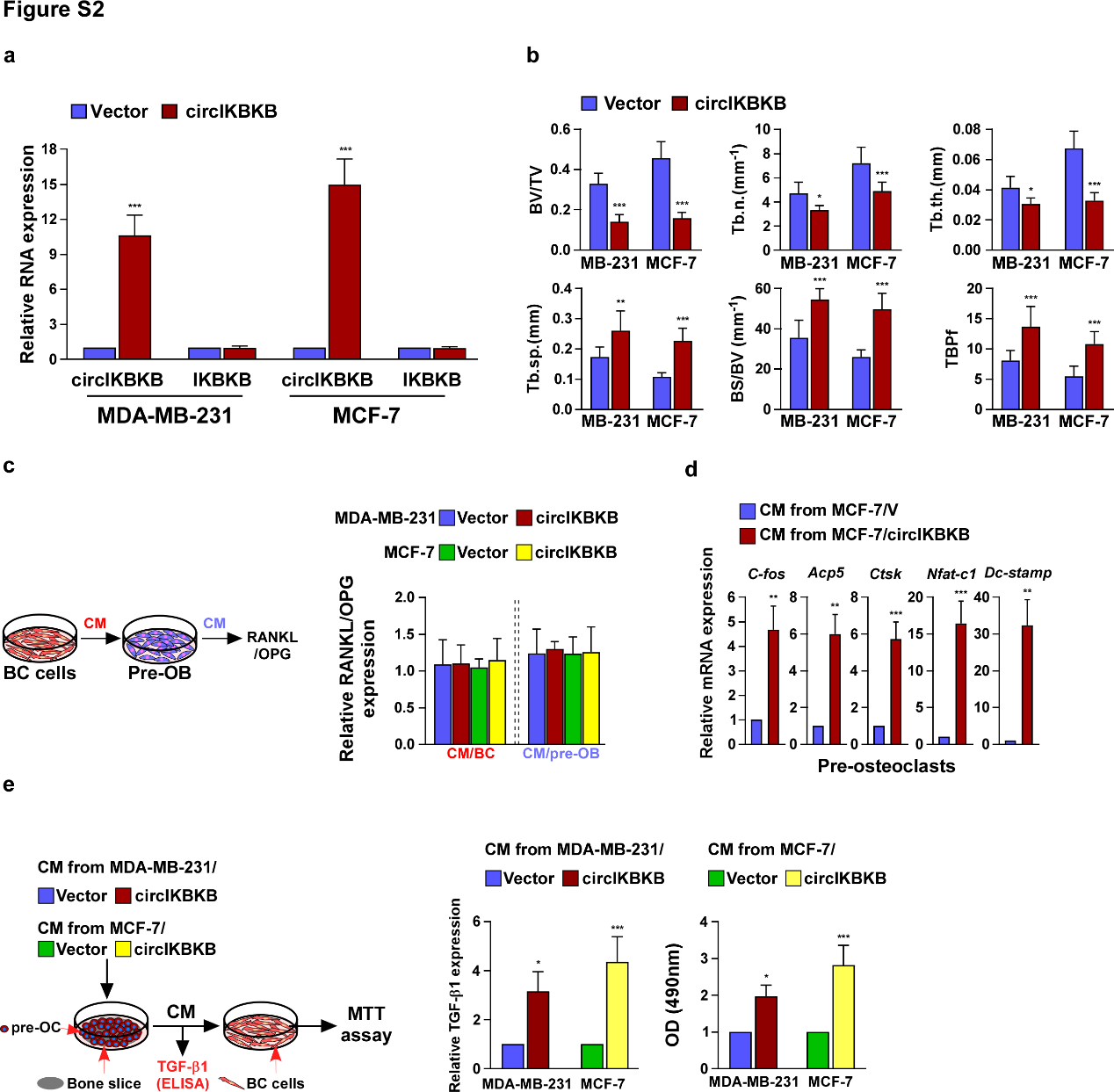
**

**Figure S2.** Overexpression of circIKBKB promotes osteolytic bone metastasis of BC *in vivo*.  **a.** qRT-PCR analysis of circIKBKB and IKBKB expression in the vector- or circIKBKB-overexpressing cells. GAPDH served as a loading control. **b**. Quantification of bone parameters from representative mice in Fig. 2b. **c**. Left: Schematic illustration of treatments of cells. Right: ELISA analysis of RANKL/OPG ratio in medium obtained from pre-osteoblasts treated by CM from the indicated BC cells. **d**. qRT-PCR analysis of mRNA level of osteoclast differentiation markers, including C-fos, Acp5, Ctsk, Nfat-c1 and Dc-stamp, in pre-osteoclasts treated by CM from the indicated BC cells. GAPDH serve as a loading control. **e**. Left: Schematic illustration of “vicious cycle” between cancer cells and osteoclasts. Middle: ELISA analysis of TGF-β1 levels in CM from pre-osteoclasts cultured onto the bone slice in the presence of CM from the indicated BC cells. Right: MTT analysis of growth curves of BC cells from experiment in the left panel. Each error bar represents the mean ± SD of three independent experiments. * *P* < 0.05, ** *P* < 0.01, *** *P* < 0.001.

**
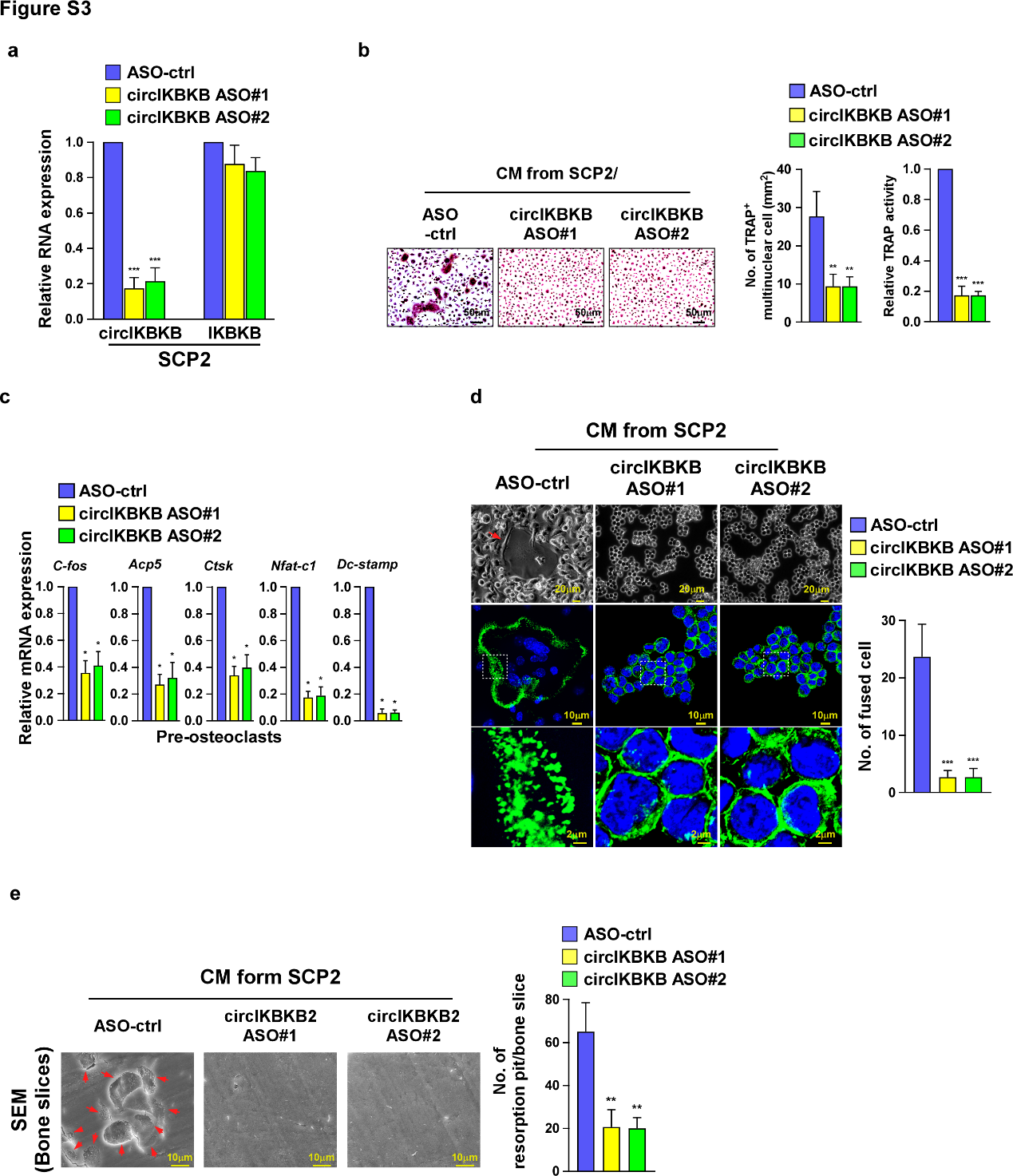
**

**Figure S3.** Silencing circIKBKB reduced capability of BC cells in inducing osteoclastogenesis. **a.** qRT-PCR analysis of circIKBKB and linear IKBKB expression in the control or circIKBKB-silenced cells. GAPDH served as a loading control. **b**. Image (left) and quantification (right) of TRAP^+^-multinuclear osteoclasts treated by CM from the indicated BC cells. **c**. qRT-PCR analysis of mRNA levels of osteoclast differentiation markers, including C-fos, Acp5, Ctsk, Nfat-c1 and Dc-stamp, in pre-osteoclasts treated by CM from the indicated BC cells. GAPDH served as a loading control. **d**. Left: Phase contrast micrograph of pre-osteoclasts treated with CM from indicated cells (upper) and IF staining images of phalloidin (F-actin) (middle and lower). Scale Bar, 20µm (upper), 10µm (middle) and 2µm (lower). Right: Quantification of number of fused multinuclear cells from experiment in the left panel. **e**. Bone resorption assay analysis of pre-osteoclasts cultured onto the bone slices treated with CM from indicated cells, then bone slices were fixed for scanning electron microscopy (SEM) (left) and quantification of the number of resorption pits per bone slice (right). Each error bar represents the mean ± SD of three independent experiments. * *P* < 0.05, ** *P* < 0.01, *** *P* < 0.001.

**
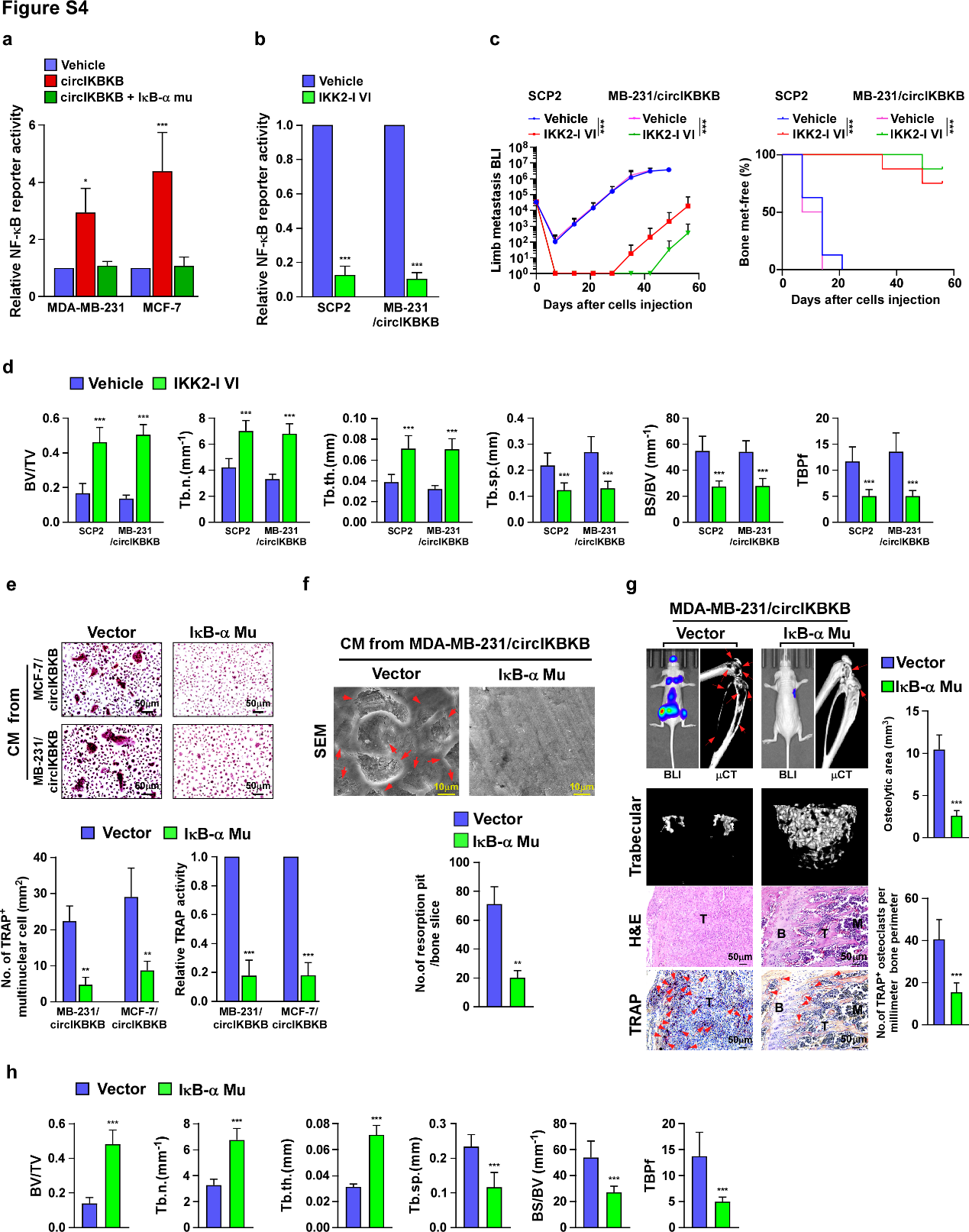
**

**Figure S4.** Activation of NF-κB is essential for circIKBKB-induced BC bone-metastasis. **a.** Relative NF-κB-driven luciferase activity was analyzed in the indicated cells treated with TNF-α (2ng/ml). **b.** Relative NF-κB-driven luciferase activity was analyzed in the indicated cells treated with TNF-α (2ng/ml) plus vehicle or IKK2-I VI (20nm/L). **c.** Normalized BLI signals of bone metastases and Kaplan-Meier bone metastasis-free survival curve of mice from indicated experimental group (n=8/group) as shown in Fig. 3h. **d.** Quantification of bone parameters from representative mice in Fig. 3h. **e.** Image (upper) and quantification (lower) of TRAP^+^-multinuclear osteoclasts treated by CM from the indicated BC cells. **f.** Image (upper) and quantification (lower) of resorption pit per bone slice treated with CM from indicated cells. **g.** Left: BLI, μCT (longitudinal and trabecular section) and histological (H&E and TRAP staining) images of bone lesions from representative mice. Scale bar, 50 μm. Right: Quantification of μCT osteolytic lesion area and TRAP^+^ osteoclasts along the bone-tumor interface of metastases from experiment in the left panel. **h.** Quantification of bone parameters from representative mice in Supplemental Fig. 3g. Each error bar represents the mean ± SD of three independent experiments. * *P* < 0.05, ** *P* < 0.01, *** *P* < 0.001.

**
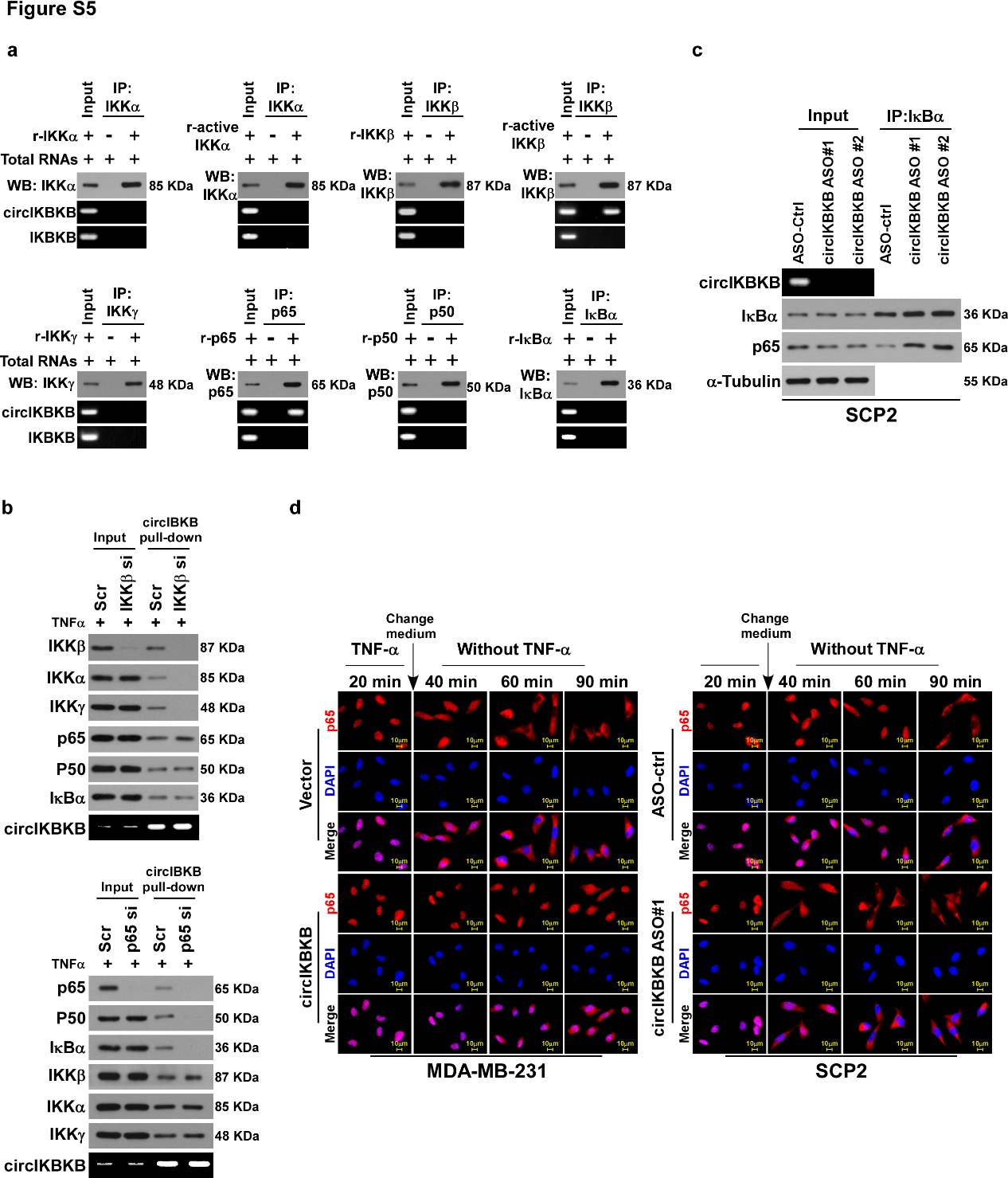
**

**Figure S5.** Cytoplasmic circIKBKB facilitates IKKβ-mediated IκBα phosphorylation and nuclear circIKBKB inhibited IκBα feedback loop.  **a.** IP assay analysis of the direct interaction of indicated components of NF-κB signaling with circIKBKB using indicated recombinant protein. **b.** RNA pull-down assay analysis of interaction of IKKα/β/γ and p65/p50/IκBα complexs with circIKBKB in cytoplasm and nuclear. ic circIKBKB facilitates IKKβ-mediated IκBα phosphorylation and nuclear circIKBKB. **c.** Co-IP assay analysis of the p65/IκB-α interaction in the control and circIKBKB-silenced cells. **d.** Immunofluorescence staining of NF-κB signal in the indicated cells treated with TNF-α (10 ng/ml) for 20 min then replaced by medium without TNF-α.

**
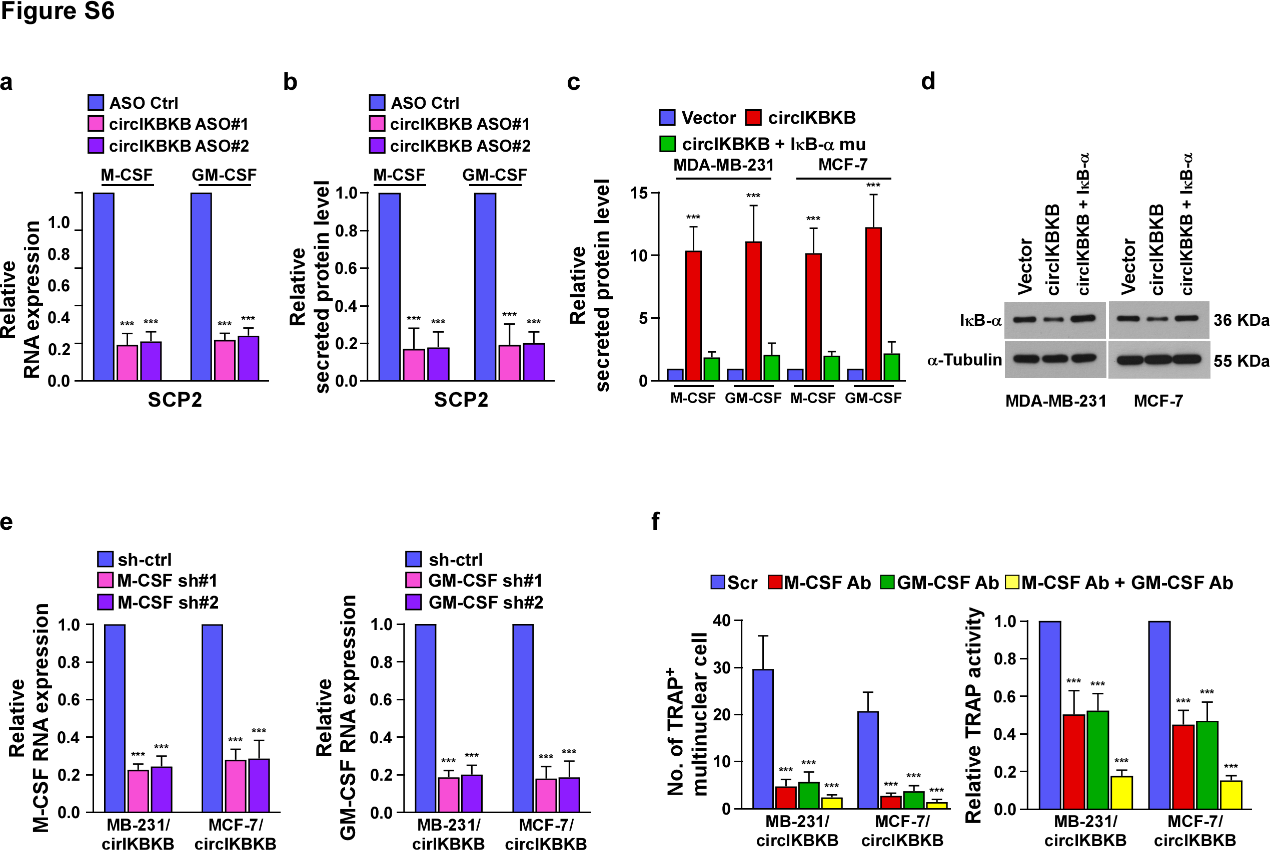
**

**Figure S6.** circIKBKB induces expression of M-CSF and GM-CSF.  **a.** qRT-PCR analysis of expression of M-CSF and GM-CSF in the control and circIKBKB-silenced cells. GAPDH served as a loading control. **b.** ELISA analysis of expression of secreted M-CSF and GM-CSF in CM from the indicated cells. **c.** ELISA analysis of expression of secreted M-CSF and GM-CSF in CM from the indicated cells. **d.** WB analysis of IκB-α expression in the indicated cells. α-tubulin served as a loading control. **e.** qRT-PCR analysis of expression of M-CSF (left) and GM-CSF (right) in the indicated cells. GAPDH served as a loading control. **f.** Quantification of TRAP^+^-multinuclear osteoclasts (left) and TRAP activity (right) in the indicated cells. Each error bar represents the mean ± SD of three independent experiments. * *P* < 0.05, ** *P* < 0.01, *** *P* < 0.001.

**
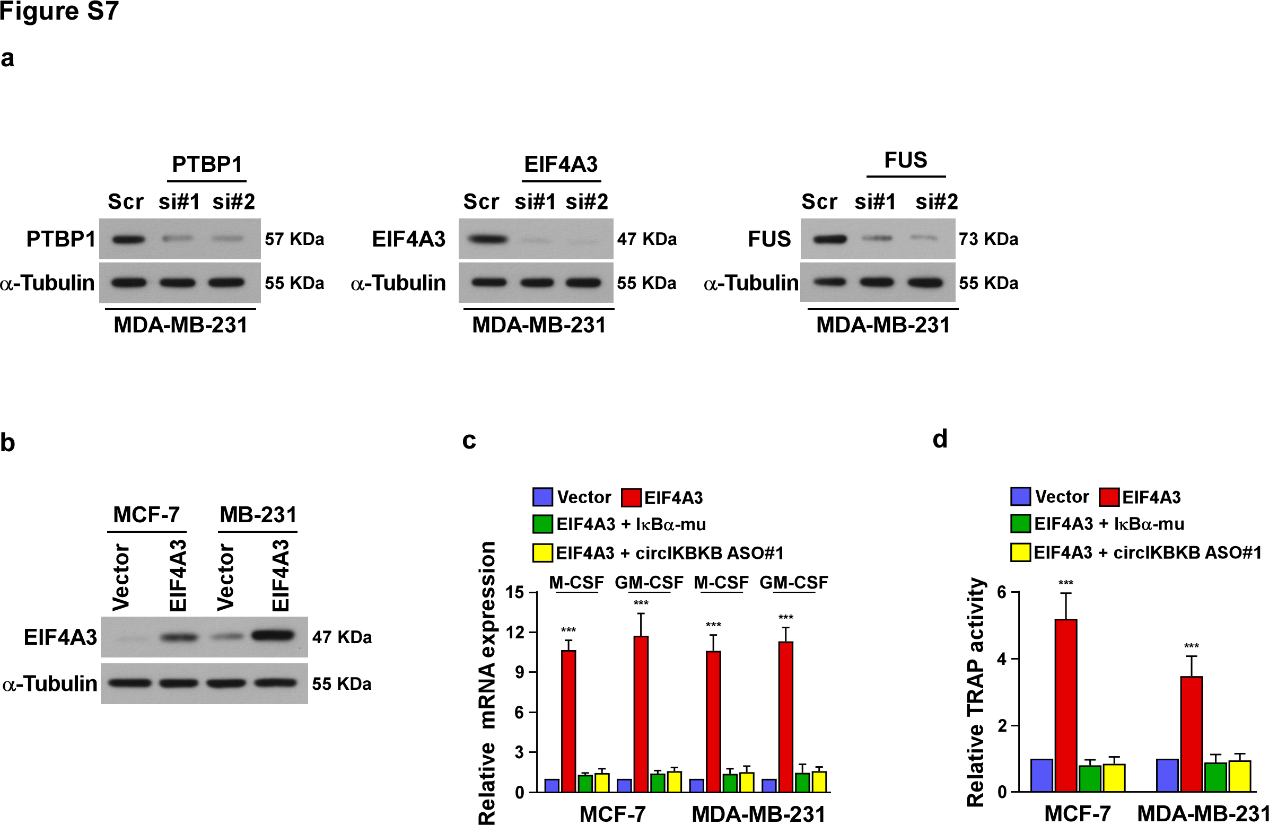
**

**Figure S7.** EIF4A3 promotes osteoclastogenesis through circIKBKB/NF-κB signaling. **a.** WB analysis of PTBP1, EIF4A3 and FUS expression in the PTBP1, EIF4A3 and FUS -silenced and control cells, respectively. α-tubulin served as a loading control. **b.** WB analysis of EIF4A3 expression in the indicated cells. α-tubulin served as a loading control. **c.** qRT-PCR analysis of expression of M-CSF and GM-CSF in the indicated cells. **d.** Quantification of TRAP activity from experiment in Fig. 6i. Each error bar represents the mean ± SD of three independent experiments. * *P* < 0.05, ** *P* < 0.01, *** *P* < 0.001.

**
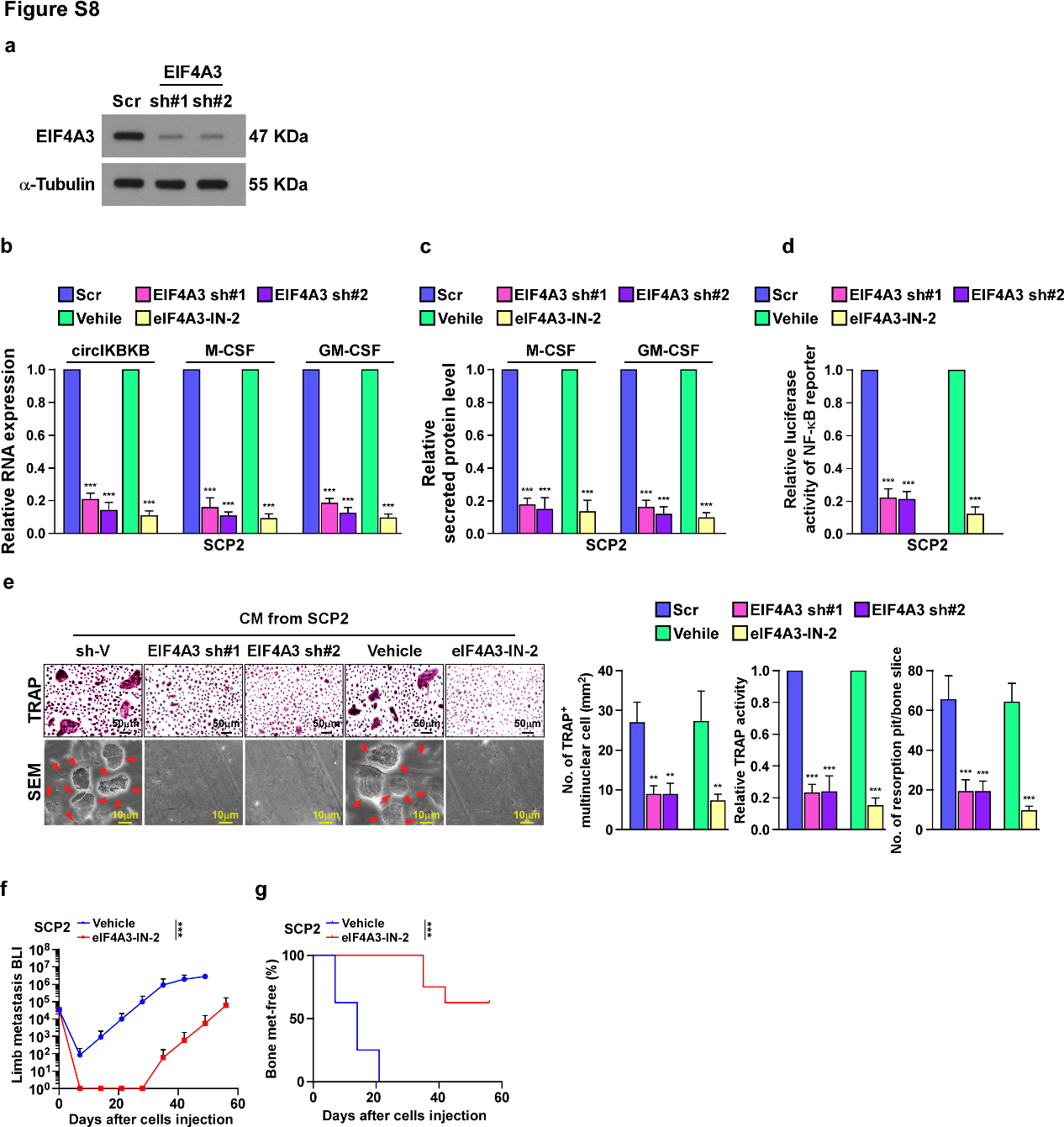
**

**Figure S8.** Blocking EIF4A3 reduced circIKBKB-induced osteoclastogenesis and bone-metastasis. **a.** WB analysis of EIF4A3 expression in the EIF4A3-silenced and control cells. α-tubulin served as a loading control. **b.** qRT-PCR analysis of expression of circIKBKB, M-CSF and GM-CSF in the indicated cells. GAPDH served as a loading control. **c.** ELISA analysis of expression of secreted M-CSF and GM-CSF in CM from the indicated cells. **d.** Relative NF-κB-driven luciferase activity was analyzed in the indicated cells treated with TNF-α (2ng/ml). **e.** Left: Image of TRAP^+^-multinuclear osteoclasts (upper) and resorption pit (lower) treated with CM from indicated cells. Right: Quantification of TRAP^+^-multinuclear osteoclasts, TRAP activity and resorption pits per bone slice from experiment in the left panel. **f-g.** Normalized BLI signals of bone metastases (**f**) and Kaplan-Meier bone metastasis-free survival curve (**g**) of mice from experiments in Fig. 7d (n = 8/group). Each error bar represents the mean ± SD of three independent experiments. * *P* < 0.05, ** *P* < 0.01, *** *P* < 0.001.

**References:**

1. Zhuang X, Zhang H, Li X, Li X, Cong M, Peng F, Yu J, Zhang X, Yang Q, Hu G. Differential effects on lung and bone metastasis of breast cancer by Wnt signalling inhibitor DKK1**.** *Nat Cell Biol* 2017, 19**:**1274-1285. https://doi.org/10.1038/ncb3613

2. Jiang L, Yu L, Zhang X, Lei F, Wang L, Liu X, Wu S, Zhu J, Wu G, Cao L, et al. miR-892b Silencing Activates NF-kappaB and Promotes Aggressiveness in Breast Cancer**.** *Cancer Res* 2016, 76**:**1101-1111. https://doi.org/10.1158/0008-5472.CAN-15-1770

3. Wu G, Song L, Zhu J, Hu Y, Cao L, Tan Z, Zhang S, Li Z, Li J. An ATM/TRIM37/NEMO Axis Counteracts Genotoxicity by Activating Nuclear-to-Cytoplasmic NF-kappaB Signaling**.** *Cancer Res* 2018, 78**:**6399-6412. https://doi.org/10.1158/0008-5472.CAN-18-2063

4. Lin C, Liu A, Zhu J, Zhang X, Wu G, Ren P, Wu J, Li M, Li J, Song L. miR-508 sustains phosphoinositide signalling and promotes aggressive phenotype of oesophageal squamous cell carcinoma**.** *Nat Commun* 2014, 5**:**4620. https://doi.org/10.1038/ncomms5620

5. Cui YM, Jiao HL, Ye YP, Chen CM, Wang JX, Tang N, Li TT, Lin J, Qi L, Wu P, et al. FOXC2 promotes colorectal cancer metastasis by directly targeting MET**.** *Oncogene* 2015, 34**:**4379-4390. https://doi.org/10.1038/onc.2014.368

6. Zhang S, Xu Y, Xie C, Ren L, Wu G, Yang M, Wu X, Tang M, Hu Y, Li Z, et al. RNF219/alpha-Catenin/LGALS3 Axis Promotes Hepatocellular Carcinoma Bone Metastasis and Associated Skeletal Complications**.** *Adv Sci (Weinh)* 2021, 8**:**2001961. https://doi.org/10.1002/advs.202001961

7. Li J, Guan HY, Gong LY, Song LB, Zhang N, Wu J, Yuan J, Zheng YJ, Huang ZS, Li M. Clinical significance of sphingosine kinase-1 expression in human astrocytomas progression and overall patient survival**.** *Clin Cancer Res* 2008, 14**:**6996-7003. https://doi.org/10.1158/1078-0432.CCR-08-0754

8. Chu C, Quinn J, Chang HY. Chromatin isolation by RNA purification (ChIRP)**.** *J Vis Exp* 2012. https://doi.org/10.3791/3912
